# Three new wood-inhabiting corticioid fungi from the Qinling Mountains, China

**DOI:** 10.3897/mycokeys.138.204396

**Published:** 2026-08-03

**Authors:** Le-Le Wan, Lan-Shuo Zhang, Jun-Hong Dong, Long Zeng, Shun Liu, Bao-Kai Cui

**Affiliations:** 1 State Key Laboratory of Efficient Production of Forest Resources, School of Ecology and Nature Conservation, Beijing Forestry University, Beijing 100083, China State Key Laboratory of Efficient Production of Forest Resources, School of Ecology and Nature Conservation, Beijing Forestry University Beijing China

**Keywords:** Macrofungi, molecular phylogeny, new taxa, Qinling Mountains, taxonomy

## Abstract

Wood-inhabiting fungi are key decomposers in forest ecosystems, but the diversity of corticioid fungi in the Qinling Mountains remains insufficiently explored. During surveys of macrofungi in Zhashui County and Shangzhou District in the Qinling Mountains, several specimens representing three undescribed taxa of corticioid fungi were collected. Based on detailed morphological examinations and phylogenetic analyses of combined ITS+nLSU datasets by using Maximum Likelihood and Bayesian Inference methods, three new species, viz. *Hymenochaete zhashuiensis*, *Hyphoderma brevicystidiatum* and *Steccherinum shangzhouense*, are described and illustrated. *Hymenochaete zhashuiensis* is characterized by effused-reflexed basidiomata with a reddish to rust-brown, cracked hymenial surface, abundant dark brown hymenial setae, the presence of dendrohyphidia, and ellipsoid to broadly ellipsoid basidiospores (4–5.6 × 2.8–3.5 µm). *Hyphoderma brevicystidiatum* is distinguished by resupinate, basidiomata white to cream with hymenial surface, clamped generative hyphae, short cylindrical to subcylindrical cystidia, subclavate to clavate basidia, and ellipsoid to subcylindrical basidiospores (6.2–10 × 2.7–4.2 µm). *Steccherinum shangzhouense* is characterized by resupinate and membranaceous basidiomata, a white to cream, tuberculate to slightly grandinioid and cracked hymenial surface, simple-septate generative hyphae, the absence of cystidia and skeletocystidia, and broadly ellipsoid to oblong ellipsoid basidiospores (5.2–7.9 × 2.5–5 µm). These findings enrich the wood-inhabiting fungal diversity in the Qinling Mountains and indicate that this region remains underexplored for wood-inhabiting fungi.

## Introduction

Wood-inhabiting fungi are a group of macrofungi primarily composed of polyporoid and corticioid fungi, in which they play a crucial role as decomposers in forest ecosystems, driving wood degradation and the recycling of organic matter (Dong et al. 2024). Among them, polypore fungi usually produce poroid basidiomata and have been well investigated in China (Wu et al. 2022; Xu et al. 2025). While corticioid fungi usually produce resupinate or effused-reflexed basidiomata, their inconspicuous basidiomata often cause these fungi to be overlooked in biodiversity surveys, and 1400 species have been reported in China (Dai et al. 2004b; Yuan et al. 2026). During surveys of macrofungi in Zhashui County and Shangzhou District of the Qinling Mountains, we collected several corticioid fungal specimens that cannot be assigned to any known species. Preliminary morphological observations indicate that these specimens belong to the genera *Hymenochaete* Lév., *Hyphoderma* Wallr. and *Steccherinum* Gray.

The genus *Hymenochaete* was established by Léveillé (1846), with *H. rubiginosa* (Dicks.) Lév. as the type species and belongs to *Hymenochaetaceae* Donk, *Hymenochaetales* Oberw. (Dai 2010; He and Dai 2012; Parmasto et al. 2014; Liu et al. 2025). The genus exhibits annual to perennial, resupinate, effused-reflexed, pileate or stipitate basidiomata, brownish to reddish brown hymenial surface, monomitic or dimitic hyphal system, simple-septate generative hyphae, hymenial setae or other setal elements, and hyaline to brownish, thin- to thick-walled basidiospores (Parmasto 2001; He and Dai 2012; Liu et al. 2025). Traditional classification discriminated species or sections mainly based on basidiomatal structure and the presence or absence of layers such as the setal layer, context or subiculum, and cortex (Burt 1918; Léger 1998). However, molecular phylogenetic studies have shown that morphological characters alone are insufficient to delimit species within this genus (Wagner and Fischer 2002; Parmasto et al. 2014; Liu et al. 2025). Subsequent molecular phylogenetic studies have clarified the phylogenetic relationships of *Hymenochaete* and related non-poroid *Hymenochaetaceae* and revealed extensive cryptic diversity worldwide (Parmasto et al. 2014; Liu et al. 2025; Wang et al. 2026). In China, some species of *Hymenochaete* have been described based on morphological characteristics and molecular phylogenetic evidence (Dai 2010; He and Dai 2012; He et al. 2017; Liu et al. 2025; Wang et al. 2026). To date, the genus *Hymenochaete* comprises approximately 265 recognized species, of which 120 have been recorded in China (Liu et al. 2025; Zhao et al. 2025; Wang et al. 2026; Xu et al. 2026; Zhu et al. 2026).

The genus *Hyphoderma* was established by Wallroth (1833), with *H. setigerum* (Fr.) Donk as the type species and belongs to *Hyphodermataceae* Jülich, *Polyporales* Gäum. (Donk 1957; Larsson 2007; Justo et al. 2017; Li et al. 2026; Yang et al. 2025). The genus exhibits considerable macromorphological diversity, with resupinate to effused-reflexed, ceraceous to membranaceous basidiomata, smooth, tuberculate, grandinioid or odontioid hymenophore, monomitic to dimitic hyphal system with clamped generative hyphae, suburniform to subcylindrical or clavate basidia, and ellipsoid to subglobose basidiospores (Bernicchia and Gorjón 2010; Guan et al. 2021; Duan et al. 2023; Yang et al. 2023). Taxonomically, the generic delimitation of *Hyphoderma* and its related genera has long been controversial. Traditional classification included a broad assemblage of corticioid fungi in *Hyphoderma*, but subsequent molecular phylogenetic studies demonstrated that the traditionally circumscribed genus was polyphyletic, leading to the transfer of several species to related genera such as *Peniophorella* P. Karst. (Larsson 2007; Justo et al. 2017; Li et al. 2026). Subsequent molecular phylogenetic studies have refined the circumscription of *Hyphoderma* and confirmed the independent phylogenetic position of the core *Hyphoderma* lineage within *Hyphodermataceae* (Justo et al. 2017; Yang et al. 2023, 2025; Li et al. 2026). In recent years, multiple new species of *Hyphoderma* have been described in China based on morphological characteristics and molecular phylogenetic evidence (Guan and Zhao 2021a, 2021b; Ma et al. 2021; Yang et al. 2023, 2025; Su et al. 2024). Currently, the genus *Hyphoderma* comprises approximately 132 species, of which 54 species are distributed in China (Guan et al. 2021; Li et al. 2026; Yuan et al. 2026).

The genus *Steccherinum* was established by Gray (1821), with *S. ochraceum* (Pers.) Gray as the type species and belongs to *Steccherinaceae* Parmasto, *Polyporales* (Zhang et al. 2025; Westphalen et al. 2026). The genus exhibits resupinate to effused-reflexed or pileate basidiomata with membranaceous to corky or waxy, and odontioid, hydnoid, grandinioid or poroid hymenophore, monomitic to dimitic hyphal system, thick-walled and encrusted skeletocystidia, and ellipsoid to subglobose basidiospores (Ryvarden 1991; Westphalen et al. 2021, 2026). Taxonomically, the generic delimitation between *Steccherinum* and its closely related genus *Junghuhnia* Corda has long been controversial (Westphalen et al. 2018). Traditional classification discriminates these two genera mainly based on hymenophoral morphology, with *Junghuhnia* characterized by a poroid hymenophore and *Steccherinum* by an odontioid hymenophore (Du et al. 2020). Subsequent molecular phylogenetic studies have verified that *Steccherinum* comprised both poroid and odontioid species, further clarifying its independent phylogenetic position within the family *Steccherinaceae* (Miettinen et al. 2012; Justo et al. 2017; Yuan et al. 2019; Westphalen et al. 2021, 2026). Recently, multiple new *Steccherinum* species have been described based on morphological characteristics and molecular phylogenetic evidence in China (Dong et al. 2022, 2023; Liu et al. 2023b; Wang et al. 2024; Zhang et al. 2025). The genus *Steccherinum* currently comprises 91 accepted species worldwide, and 29 *Steccherinum* taxa have been published or recorded from China (Yuan and Wu 2012; Liu and Dai 2021; Dong et al. 2022; Yuan et al. 2026).

To further clarify the taxonomic status of these specimens, species identifications were performed based on detailed morphological observations and phylogenetic analyses of the combined ITS+nLSU dataset. The results indicate that these specimens represent three new species belonging to *Hymenochaete*, *Hyphoderma*, and *Steccherinum*, respectively.

## Materials and methods

### Morphological studies

Fresh specimens of *Hymenochaete*, *Hyphoderma* and *Steccherinum* were collected from Zhashui County and Shangzhou District, Shaanxi Province, China. The basidiomata were photographed *in situ*, and macromorphological characters, substrates and habitats were recorded in the field. The specimens were dried and deposited at the herbarium of the Institute of Microbiology, Beijing Forestry University (**BJFC**), Beijing, China. Color terms used in macromorphological descriptions follow Kornerup and Wanscher (1967).

Macromorphological descriptions were based on field notes and laboratory observations. Micromorphological characters, measurements and drawings were made from slide preparations mounted in 5% potassium hydroxide (KOH), Cotton Blue (CB) and Melzer’s reagent (IKI), using a Nikon Eclipse 80i microscope (Nikon, Tokyo, Japan). At least 30 basidiospores were measured from each specimen. Sterigmata were excluded from basidial measurements, and the hilar appendage was excluded from basidiospore measurements. The following abbreviations are used: **CB** = Cotton Blue, **CB–** = acyanophilous, **IKI** = Melzer’s reagent, **IKI–** = neither amyloid nor dextrinoid, **KOH** = 5% potassium hydroxide, **L** = mean basidiospore length, **W** = mean basidiospore width, **Q** = range of the length/width ratio of basidiospores, **Qm** = mean length/width ratio of basidiospores, and **n** = a/b, where a represents the number of measured structures and b represents the number of specimens examined. When sufficient measurements were available, 5% of measurements were excluded from each end of the range and are given in parentheses. The detailed morphological methods followed Sun et al. (2022) and Liu et al. (2023a).

### DNA extraction and sequencing

Genomic DNA was extracted from dried specimens using the CTAB rapid plant genome DNA extraction kit DN14 (Aidlab Biotechnologies, Beijing, China), following the manufacturer’s instructions with modifications as described by Song et al. (2025). The internal transcribed spacer region (ITS) was amplified with primers ITS5 and ITS4 (White et al. 1990), and the nuclear large subunit ribosomal RNA gene region (nLSU) was amplified with primers LR0R and LR7 (Hopple Jr and Vilgalys 1999).

The polymerase chain reaction (PCR) program for ITS consisted of an initial denaturation at 95 °C for 3 min, followed by 35 cycles at 94 °C for 40 s, 56 °C for 45 s and 72 °C for 1 min, and a final extension at 72 °C for 10 min. The PCR program for nLSU consisted of an initial denaturation at 94 °C for 1 min, followed by 35 cycles at 94 °C for 30 s, 50 °C for 1 min and 72 °C for 90 s, and a final extension at 72 °C for 10 min. PCR products were sequenced at the Beijing Genomics Institute (BGI), China, using the same primers.

The newly generated sequences were assembled and edited, and then deposited in GenBank. All newly generated sequences and reference sequences used in the phylogenetic analyses are listed in Table 1.

**Table 1.** Taxa information and GenBank accession numbers of sequences used in this study.

**SPECIES NAME**	**SPECIMEN VOUCHER**	**ITS**	**nLSU**	**REFERENCE**
*Cabalodontia delicata*	MCW 693/19	MT849297	MT849297	Westphalen et al. 2021
*Diplomitoporus crustulinus*	FD-137	KP135299	KP135211	Justo et al. 2017
*Hydnoporia olivacea*	Miettinen X3273	MK514610	–	Miettinen et al. 2019
*H. olivacea*	Miettinen X3403	MK514612	–	Miettinen et al. 2019
*Hymenochaete acanthophysata*	CBS 925.96	MH862623	AF385144	Vu et al. 2019; Wagner and Fischer 2002
*H. acerosa*	He 344	NR_120042	–	He and Li 2011
*H. adhaerens*	Spirin 4994	KM017411	–	Spirin et al. 2015
*H. adhaerens*	Spirin 6246	KM017412	–	Spirin et al. 2015
*H. adnata*	He 560	–	KU975527	Liu et al. 2025
*H. adnata*	He 951	–	KU975528	Liu et al. 2025
*H. adnata*	He 655	OR287568	OR287643	Liu et al. 2025
*H. adusta*	He 207	JQ279523	KU975497	Nie et al. 2017
*H. alpina*	He 1388	OR287604	OR287674	Liu et al. 2025
*H. alpina*	He 4918	OR287605	OR287675	Liu et al. 2025
*H. angustispora*	Dai 17045	MF370592	MF370598	He et al. 2017
*H. angustispora*	Dai 17049	MF370593	MF370599	He et al. 2017
*H. anomala*	He 1276	OR287531	OR287616	Liu et al. 2025
*H. anomala*	He 592	JQ279566	JQ279650	He and Dai 2012
*H. asetosa*	Dai 10756	JQ279559	JQ279642	He and Dai 2012
*H. asiatica*	He 1270	OR287593	OR287663	Liu et al. 2025
*H. asiatica*	He 5800	OR287594	–	Liu et al. 2025
*H. atrobrunnea*	Dai 18810	OR287608	OR287656	Liu et al. 2025
*H. atrobrunnea*	Dai 18821	OR287591	OR287657	Liu et al. 2025
*H. attenuata*	He 28	JQ279526	JQ279633	He and Dai 2012
*H. australis*	TAAM171362	KM017414	HE650990	Spirin et al. 2015; Parmasto et al. 2014
*H. austrosinensis*	He 1009	OR287589	OR287660	Liu et al. 2025
*H. austrosinensis*	He 7078	OR287590	OR287661	Liu et al. 2025
*H. bannaensis*	CLZhao 35721	PQ847494	PQ847499	Deng et al. 2025
*H. baishanzuensis*	Wei 11432	PV883105	–	Zhu et al. 2026
*H. baishanzuensis*	Wei 11406	PV883104	–	Zhu et al. 2026
*H. bambusicola*	He 4116	KY425674	KY425681	Nie et al. 2017
*H. berteroi*	He 1488	KU975459	KU975498	He et al. 2017
*H. biformisetosa*	He 1445	KF908247	KU975499	Yang and He 2014; He еt al. 2017
*H. bispora*	He 4993	OR287532	OR287617	Liu et al. 2025
*H. boddingii*	MEH-69996	MN030341	MN030347	Rossi et al. 2020
*H. boidinii*	CBS 765.91	MH862335	–	Vu et al. 2019
*H. borbonica*	CBS 731.86	MH862026	MH873716	Vu et al. 2019
*H. brunnea*	He 581	OR287582	OR287655	Liu et al. 2025
*H. brunnea*	He 591	OR287581	OR287654	Liu et al. 2025
*H. campylopora*	Cui 7393	JQ279513	JQ279629	He et al. 2017
*H. cana*	He 1305	KF438169	KF438172	He and Li 2014
*H. chimonobambusae*	HMZhou 710	PV475582	PV765708	Wang et al. 2026
*H. cervinoidea*	CBS 736.86	MH862027	–	Vu et al. 2019
*H. cinerea*	He 1432	OR287602	OR287672	Liu et al. 2025
*H. cinerea*	He 1483	OR287603	OR287673	Liu et al. 2025
*H. cinereoalba*	CLZhao 10420	OR287566	–	Liu et al. 2025
*H. cinereoalba*	CLZhao 4252	OR287567	OR287642	Liu et al. 2025
*H. cinereoalba*	He 6162	OR287559	OR287636	Liu et al. 2025
*H. cinnamomea*	He 2074	KU975460	KU975500	Nie et al. 2017
*H. cinnamomea*	He 755	JQ279548	JQ279658	He and Dai 2012
*H. coffeana*	He 250	OR287533	–	Liu et al. 2025
*H. colliculosa*	Dai 16428	MF370596	MF370603	He et al. 2017
*H. conchata*	LWZ 20140728-13	KX258960	–	Pan and Zhou 2016
*H. conifericola*	He 779	JQ279538	JQ279641	Liu et al. 2025
*H. conifericola*	He 788	JQ279539	OR287666	Liu et al. 2025
*H. contiformis*	He 1166	KU975461	KU975501	He et al. 2017
*H. cruenta*	He 766	JQ279595	JQ279681	He et al. 2017
*H. cruenta*	SFC20170811_12	MT044423	–	Park et al. 2020
*H. curtisii*	USDA FP-103876-Sp	–	HE650996	Parmasto et al. 2014
*H. curtisii*	He 2061	KU975462	KU975502	Liu et al. 2025
*H. cylindrospora*	Dai 14890	OR287562	–	Liu et al. 2025
*H. cylindrospora*	He 773	OR287563	OR287638	Liu et al. 2025
*H. damicornis*	URM 84261	KC348466	–	Du et al. 2021
*H. denticulata*	CBS 780.91	MH862336	AF385155	Vu et al. 2019
*H. dichotoma*	Dai 18649	OR287573	OR287647	Liu et al. 2025
*H. dichotoma*	Dai 18779	OR287574	OR287648	Liu et al. 2025
*H. dracaenicola*	Dai 22090	MW559797	MW559802	Du et al. 2021
*H. dracaenicola*	Dai 22096	MW559798	MW559803	Du et al. 2021
*H. duportii*	AFTOL-ID 666	DQ404386	AY635770	Matheny et al. 2006
*H. epichlora*	He 524	OR287534	OR287618	Liu et al. 2025
*H. epichlora*	He 525	JQ279549	JQ279659	He and Dai 2012
*H. erastii*	He 1639	OR287569	OR287644	Liu et al. 2025
*H. erastii*	He 1644	OR287570	–	Liu et al. 2025
*H. fissurata*	CLZhao 860	MG231569	–	GenBank
*H. fissurata*	He 1193	OR287535	OR287619	Liu et al. 2025
*H. flava*	He 278	OR287586	–	Liu et al. 2025
*H. flava*	He 346	OR287587	OR287658	Liu et al. 2025
*H. flava*	He 855	OR287588	OR287659	Liu et al. 2025
*H. floridea*	He 529	JQ279598	KU975505	He and Dai 2012; Liu et al. 2025
*H. fuliginosa*	He 1188	KU975465	KU975506	He et al. 2017
*H. fuliginosa*	He 2077	KU975468	KU975510	Liu et al. 2025
*H. fuliginosa*	UC2023222	KP814513	–	GenBank
*H. fulva*	He 640	JQ279565	JQ279648	He and Dai 2012
*H. globispora*	He 911	OR287536	KU975508	Liu et al. 2025
*H. granulata*	He 1472	KU975495	KU975547	Liu et al. 2025
*H. hainanensis*	He 1509	OR287595	OR287664	Liu et al. 2025
*H. hainanensis*	He 1534	OR287596	OR287665	Liu et al. 2025
*H. huangshanensis*	He 432	JQ279533	JQ279671	He and Dai 2012
*H. huangshanensis*	He 441	JQ279535	JQ279669	He and Dai 2012
*H. hubeiensis*	Dai 17959	OR287611	OR287679	Liu et al. 2025
*H. hubeiensis*	He 5084	OR287610	OR287678	Liu et al. 2025
*H. hydnoides*	He 1309	KU975467	KU975509	Liu et al. 2025
*H. iliensis*	Yuan 374	PP769636	–	Liu et al. 2025
*H. iliensis*	Yuan 386	PP769637	–	Liu et al. 2025
*H. innexa*	He 505	OR287537	OR287620	Liu et al. 2025
*H. koeljalgii*	TAAM 159464	–	HE651003	Parmasto et al. 2014
*H. legeri*	He 1021	OR287538	OR287621	Liu et al. 2025
*H. legeri*	He 960	KU975469	KU975511	He et al. 2017
*H. leveillei*	CLZhao 10443	OR287547	–	Liu et al. 2025
*H. leveillei*	He 1437	OR287548	OR287628	Liu et al. 2025
*H. leveillei*	He 6202	OR287549	OR287629	Liu et al. 2025
*H. lictor*	He 20140723-2	KU975470	KU975512	Liu et al. 2025
*H. liyiriana*	ARFR260	OR584222	OR569028	Tepin and Singh 2025
*H. liyiriana*	ARFR260a	PQ182942	–	Tepin and Singh 2025
*H. longispora*	He 217	JQ279537	KU975514	He and Dai 2012; Nie et al. 2017
*H. luteomarginata*	He 1457	OR287614	OR287680	Liu et al. 2025
*H. luteomarginata*	He 1849	OR287615	OR287681	Liu et al. 2025
*H. macrochloae*	ARAN-Fungi 7079	MF990738	MF990743	Crous et al. 2017
*H. macrospora*	Dai 488	KM017413	–	Spirin et al. 2015
*H. major*	CLZhao 5136	OR287558	–	Liu et al. 2025
*H. major*	He 1942	KU975489	KU975541	Liu et al. 2025
*H. megaspora*	He 302	JQ279553	JQ279660	He and Dai 2012
*H. megaspora*	He 328	JQ279554	–	He and Dai 2012
*H. membranacea*	He 1899	KU975491	KU975543	Liu et al. 2025
*H. membranacea*	He 1903	KU975492	KU975544	Liu et al. 2025
*H. microcycla*	CBS 311.39	MH856027	–	Vu et al. 2019
*H. microcycla*	Cui 8555	KT283049	JQ279640	Ariyawansa et al. 2015; He and Dai 2012
*H. microcycla*	LWZ 20140719-11	KT283050	–	Ariyawansa et al. 2015
*H. micropora*	Cui 8057	JQ279518	–	He and Dai 2012
*H. minor*	He 933	JQ279555	JQ279654	He and Dai 2012
*H. minuscula*	He 253	JQ279546	KU975516	He and Dai 2012; Nie et al. 2017
*H. moniliformis*	He 1321	OR287556	OR287634	Liu et al. 2025
*H. moniliformis*	He 916	OR287555	OR287633	Liu et al. 2025
*H. montana*	He 1172	OR287606	OR287676	Liu et al. 2025
*H. montana*	He 1380	OR287607	OR287677	Liu et al. 2025
*H. mougeotii*	CBS 289.54	MH857337	MH868878	Vu et al. 2019
*H. murina*	He 569	JQ716406	JQ716412	He and Li 2013a
*H. muroiana*	He 172	JQ279541	–	He and Dai 2012
*H. muroiana*	He 405	JQ279542	KU975517	He and Dai 2012; Nie et al. 2017
*H. nanospora*	CBS 924.96	MH862622	MH874244	Vu et al. 2019
*H. nanospora*	He 475	JQ279531	JQ279672	He and Dai 2012
*H. niveomarginata*	Dai 24611	PP769638	PP769648	Liu et al. 2025
*H. niveomarginata*	He 1611	PP769639	–	Liu et al. 2025
*H. nothofagicola*	He 503	JQ279530	JQ279632	He and Dai 2012
*H. ochromarginata*	Cui 8197	JQ279578	–	He and Dai 2012
*H. ochromarginata*	He 47	JQ279579	JQ279666	He and Dai 2012
*H. odontoides*	Dai 11635	JQ279563	JQ279647	He and Dai 2012
***H. odontoides***	**Cui 24840**	**PZ458970**	**PZ466728**	**This study**
*H. orientalis*	He 4601	KY425677	KY425685	Nie et al. 2017
*H. parmastoi*	He 367	JQ780061	–	He and Li 2012
*H. paucisetigera*	Cui 7845	JQ279560	JQ279644	He and Dai 2012
*H. peroxydata*	JMB2056	KF371645	KF371648	Baltazar et al. 2014
*H. piceae*	He 1200	OR287572	OR287646	Liu et al. 2025
*H. piceae*	He 1203	OR287571	OR287645	Liu et al. 2025
*H. pinnatifida*	He 2193	KU975472	KU975519	Liu et al. 2025
*H. puerensis*	He 692	OR287612	–	Liu et al. 2025
*H. puerensis*	He 708	OR287613	–	Liu et al. 2025
*H. punctata*	HMZhou 16	PV475579	PV765709	Wang et al. 2026
*H. quercicola*	He 373	KU975474	KU975521	He et al. 2017
*H. quercicola*	He 377	OR287542	–	Liu et al. 2025
*H. ramicola*	He 1703	KU975493	KU975545	Liu et al. 2025
*H. ramicola*	He 1897	KU975494	KU975546	Liu et al. 2025
*H. ramicola*	He 5437	OR287583	–	Liu et al. 2025
*H. resupinata*	TU 100039	–	HE650988	Parmasto et al. 2014
*H. rhabarbarina*	He 280	JQ279574	KY425688	He and Dai 2012; Nie et al. 2017
*H. rhabarbarina*	LY L527	–	HE651007	Parmasto et al. 2014
*H. rheicolor*	He 2192	KU975475	KU975522	Liu et al. 2025
*H. rhododendricola*	He 392	JQ279576	–	He and Dai 2012
*H. rubiginosa*	Miettinen X3422	MK757158	–	GenBank
*H. rubiginosa*	S58	FJ820546	–	Fröhlich-Nowoisky et al. 2009
*H. rubrobrunnea*	He 1982	OR287579	KU975549	Liu et al. 2025
*H. rubrobrunnea*	He 5315	OR287578	–	Liu et al. 2025
*H. rubrobrunnea*	He 5346	OR287580	OR287653	Liu et al. 2025
*H. rufomarginata*	He 1489	KU975477	KU975524	He et al. 2017
*H. sanguinaria*	HMZhou 995	PV475583	PV765710	Wang et al. 2026
*H. senatoumbrina*	He 2437	KU975478	KU975525	Liu et al. 2025
*H. senatoumbrina*	He 4940	OR457649	OR452887	Liu et al. 2025
*H. separabilis*	BDC iNaturalist#192495191	PQ509880	–	GenBank
*H. separabilis*	He 1479	KU975479	KU975526	Liu et al. 2025
*H. separabilis*	PDD 119545	OL709441	–	GenBank
*H. separata*	He 934	OR287544	OR287626	Liu et al. 2025
*H. setipora*	Cui 6301	JQ279515	JQ279639	He and Dai 2012
*H. setipora*	LE-BIN 3006	PQ361605	–	GenBank
*H. setipora*	SL1822	OR636140	–	GenBank
*H. setulohypha*	He 5570	OR287561	OR287637	Liu et al. 2025
*H. setulohypha*	He 5602	OR287560	–	Liu et al. 2025
*H. sharmae*	66088	MK588753	MK588836	Wang et al. 2019
*H. sharmae*	CAL 1535	KY929017	KY929018	Wang et al. 2019
*H. sichuanensis*	He 1340	OR287577	OR287641	Liu et al. 2025
*H. sichuanensis*	He 1385	OR287585	OR287652	Liu et al. 2025
*H. sinensis*	CLZhao 26040	OR659001	PP425893	Li et al. 2024
*H. sinensis*	CLZhao 26652	PQ060540	–	Li et al. 2024
*H. spathulata*	He 685	JQ279591	KU975529	He and Dai 2012; He et al. 2017
*H. sphaericola*	He 303	JQ279599	JQ279684	He and Dai 2012
*H. sphaerospora*	He 715	JQ279594	KU975531	He and Dai 2012; He et al. 2017
*H. stereoidea*	CLZhao 4321	OR287609	–	Liu et al. 2025
*H. stereoidea*	He 1661	KU975488	KU975540	Liu et al. 2025
*H. stratura*	HHB-19391	MW740246	–	Liu et al. 2025
*H. subepichlora*	He 556	OR287554	OR287632	Liu et al. 2025
*H. subepichlora*	He 559	OR287553	OR287631	Liu et al. 2025
*H. subepichlora*	He 716	PP769642	–	Liu et al. 2025
*H. subferruginea*	He 1598	KU975481	–	GenBank
*H. subfissurata*	Dai 24358A	PP769643	PP769650	Liu et al. 2025
*H. subfissurata*	He 5573	PP769644	PP769651	Liu et al. 2025
*H. subinnexa*	He 1007	OR287598	OR287668	Liu et al. 2025
*H. subinnexa*	He 1213	OR287597	OR287667	Liu et al. 2025
*H. subluteobadia*	He 10	JQ279568	OR287662	Liu et al. 2025
*H. subluteobadia*	He 8	JQ279569	KU975515	He and Dai 2012; Nie et al. 2017
*H. subporioides*	Cui 10163	KT283051	OR287627	Ariyawansa et al. 2015; Liu et al. 2025
*H. subrhabarbarina*	He 737	OR287575	OR287649	Liu et al. 2025
*H. subrhabarbarina*	He 860	OR287576	–	Liu et al. 2025
*H. subrhabarbarina*	LY L690	–	HE650994	Parmasto et al. 2014
*H. tasmanica*	He 449	JQ279582	JQ279663	He and Dai 2012
*H. tasmanica*	He 455	JQ279583	JQ279664	He and Dai 2012
*H. tongbiguanensis*	He 1552	KF908248	KU975532	Yang and He 2014; Nie et al. 2017
*H. tropica*	He 661	JQ279588	–	He and Dai 2012
*H. unicolor*	He 468a	JQ279551	JQ279662	He and Dai 2012
*H. ustulata*	He 104	JQ780066	–	He and Li 2013b
*H. vaginata*	He 2558	KU975483	KU975535	Liu et al. 2025
*H. vaginata*	He 2599	KU975484	KU975536	Liu et al. 2025
*H. variabilis*	Dai 19779	OR287584	OR287651	Liu et al. 2025
*H. variabilis*	He 1610	OR287592	OR287650	Liu et al. 2025
*H. verruculosa*	Dai 17047	–	MF370600	He et al. 2017
*H. villosa*	He 1739	KU975485	KU975537	Liu et al. 2025
*H. vitellina*	CLZhao 17846	OR287550	–	Liu et al. 2025
*H. vitellina*	He 5061	OR287551	OR287630	Liu et al. 2025
*H. vitellina*	He 627	OR287552	–	Liu et al. 2025
*H. vivida*	He 2040	OR287601	OR287671	Liu et al. 2025
*H. vivida*	He 4986	OR287600	OR287670	Liu et al. 2025
*H. vivida*	He 5288	OR287599	OR287669	Liu et al. 2025
*H. xerantica*	Cui 9209	JQ279519	JQ279635	He and Dai 2012
*H. xerantica*	E060	LC520144	LC520190	Kobayashi et al. 2020
*H. yaoshanensis*	CLZhao 20661	PP356585	–	Xu et al. 2026
*H. yaoshanensis*	CLZhao 20626	PP356584	PP785350	Xu et al. 2026
*H. yunnanensis*	He 709	OR287546	–	Liu et al. 2025
***H. zhashuiensis***	**Cui 24846**	**PZ458971**	**PZ466729**	**This study**
*Hyphoderma alboarachnum*	CLZhao 30488	PV470563	–	Li et al. 2026
*H. amoenum*	USO 286622	HE577030	–	Tellería et al. 2012
*H. asianum*	CLZhao 18091	OR141726	PP826262	Yang et al. 2025
*H. assimile*	CBS:125852	MH863808	MH875272	Vu et al. 2019
*H. australosetigerum*	MA:Fungi:92235	MN963764	–	Boonmee et al. 2021
*H. australosetigerum*	MA:Fungi:92240	MN963760	–	Boonmee et al. 2021
*H. bambusinum*	CLZhao 29903	PV469674	PV819428	Li et al. 2026
***H. brevicystidiatum***	**Cui 24844**	**PZ458969**	**PZ466727**	**This study**
***H. brevicystidiatum***	**Cui 24841**	**PZ458968**	**PZ466726**	**This study**
*H. cinereofuscum*	CLZhao 30283	PQ492372	PQ511129	Li et al. 2025
*H. cinereofuscum*	CLZhao 30341	PQ492371	PQ511128	Li et al. 2025
*H. cremeoalbum*	CLZhao 17007	OM985716	OM985753	Duan et al. 2023
*H. cremeoalbum*	NH 11538 (GB)	DQ677492	DQ677492	Larsson 2007
*H. crystallinum*	CLZhao 9338	MW917161	MW913414	Guan and Zhao 2021a
*H. crystallinum*	CLZhao 9374	MW917162	MW913415	Guan and Zhao 2021a
*H. definitum*	NH 12266 (GB)	DQ677493	DQ677493	Larsson 2007
*H. fissuratum*	CLZhao 6726	MT791330	MT791334	Ma et al. 2021
*H. fissuratum*	CLZhao 6731	MT791331	–	Ma et al. 2021
*H. floccosum*	CLZhao 17129	MW301683	MW293733	Guan and Zhao 2021b
*H. floccosum*	CLZhao 17215	MW301687	MW293735	Guan and Zhao 2021b
*H. fragilissimum*	CLZhao 17245	PV147166	PV185848	Wijesinghe et al. 2025
*H. fulgens*	CLZhao 30254	PV469670	PV819427	Li et al. 2026
*H. fulgens*	CLZhao 36600	PV829546	PV810096	Li et al. 2026
*H. fulgens*	CLZhao 37429	PV829544	PV810095	Li et al. 2026
*H. fulgens*	CLZhao 39474	PV829547	PV810098	Li et al. 2026
*H. grandineum*	CLZhao 30046	PV470561	PV819429	Li et al. 2026
*H. grandineum*	CLZhao 43328	PV470562	PV819430	Li et al. 2026
*H. granuliferum*	5273	JN710545	JN710545	Yurchenko and Wu 2014a
*H. guangdongense*	CLZhao 12657	PP235513	PP235514	Su et al. 2024
*H. incrustatum*	KHL6685	–	AY586668	Yurchenko and Wu 2014a
*H. laceratum*	CLZhao 34242	PV829552	PV810101	Li et al. 2026
*H. laceratum*	CLZhao 34672	PV829553	PV810103	Li et al. 2026
*H. laceratum*	CLZhao 34958	PV829556	PV810106	Li et al. 2026
*H. laceratum*	CLZhao 34961	PV829557	–	Li et al. 2026
*H. litschaueri*	FP-101740-Sp	KP135295	KP135219	Floudas and Hibbett 2015
*H. litschaueri*	NH 7603 (GB)	DQ677496	DQ677496	Larsson 2007
*H. macaronesicum*	MA:Fungi:90388	KC984327	–	Tellería et al. 2012
*H. macaronesicum*	TFC:Mic 15115	HE577011	–	Yurchenko and Wu 2014b
*H. marginatum*	CLZhao 3404	OM985717	OM985754	Duan et al. 2023
*H. medioburiense*	FD-335	KP135298	KP135220	Floudas and Hibbett 2015
*H. membranaceum*	CLZhao 5844	MW917167	MW913420	Guan and Zhao 2021a
*H. membranaceum*	CLZhao 6971	MW917168	MW913421	Guan and Zhao 2021a
*H. microporoides*	CLZhao 6857	MW917169	MW913422	Guan and Zhao 2021a
*H. microporoides*	CLZhao 8695	MW917170	MW913423	Guan and Zhao 2021a
*H. moniliforme*	Wu 0211-42	KC928282	–	Yurchenko and Wu 2015
*H. moniliforme*	Wu 0211-46	KC928284	–	Yurchenko and Wu 2015
*H. mopanshanense*	CLZhao 6449	OM985720	OM985759	Duan et al. 2023
*H. mopanshanense*	CLZhao 6498	MT791329	MT791333	Ma et al. 2021
*H. nemorale*	TNM F3931	KJ885183	KJ885184	Yurchenko and Wu 2015
*H. nemorale*	Wu 9508-14	KC928280	KC928281	Yurchenko and Wu 2015
*H. nitidum*	CLZhao 37195	PV470533	–	Yuan et al. 2026
*H. nitidum*	CLZhao 37232	PV470534	–	Yuan et al. 2026
*H. niveomarginatum*	CLZhao 25078	OR141728	OR506179	Yang et al. 2023
*H. nudicephalum*	CLZhao 17839	OM985721	OM985760	Duan et al. 2023
*H. nudicephalum*	Wu9307_29	AJ534269	–	Nilsson et al. 2003
*H. obtusiforme*	KHL11105	JN572910	–	Yurchenko and Wu 2014b
*H. obtusiforme*	KHL1464	JN572909	–	Yurchenko and Wu 2014b
*H. obtusum*	JS17804	–	AY586670	Yurchenko and Wu 2014b
*H. occidentale*	KHL 8477 (GB)	DQ677499	DQ677499	Larsson 2007
*H. paramacaronesicum*	MA:Fungi:87736	KC984399	–	Martín et al. 2018
*H. paramacaronesicum*	MA:Fungi:87737	KC984405	–	Martín et al. 2018
*H. pinicola*	Wu 0108-32	KJ885181	KJ885182	Yurchenko and Wu 2014b
*H. pinicola*	Wu 0108-36	KC928278	KC928279	Yurchenko and Wu 2014b
*H. prosopidis*	ARIZ HHB 8479	HE577029	–	Yurchenko and Wu 2015
*H. puerense*	CLZhao 9476	MW443045	–	Guan et al. 2021
*H. puerense*	CLZhao 9583	MW443046	MW443051	Guan et al. 2021
*H. punctatum*	CLZhao 33051	PV469675	–	Wijesinghe et al. 2025
*H. qujingense*	CLZhao 26018	OR141729	PP826263	Yang et al. 2025
*H. roseocremeum*	NH 10545	–	AY586672	Yurchenko and Wu 2014a
*H. sibiricum*	Kotiranta 22801	MF319069	MF318925	GenBank
*H. setigerum*	FCUG 1200	AJ534273	–	Nilsson et al. 2003
*H. setigerum*	FCUG 1688	AJ534272	–	Nilsson et al. 2003
*H. sinense*	CLZhao 17811	MW301682	MW293732	Guan and Zhao 2021b
*H. sinense*	CLZhao 7963	MW301679	MW293730	Guan and Zhao 2021b
*H. sordidum*	CLZhao 27379	OR141731	–	Yang et al. 2023
*H. sordidum*	CLZhao 27390	OR141732	OR506180	Yang et al. 2023
*H. subsetigerum*	HHB11620	GQ409521	–	Yurchenko and Wu 2014a
*H. subtestaceum*	CBS:125877	MH864081	MH875539	Yang et al. 2023
*H. tenuissimum*	CLZhao 16210	MW443050	MW443055	Guan et al. 2021
*H. tenuissimum*	CLZhao 7221	MW443049	MW443054	Guan et al. 2021
*H. tongbiguanense*	CLZhao 39575	PV470532	–	Yuan et al. 2026
*H. tongbiguanense*	CLZhao 39574	PV470531	–	Yuan et al. 2026
*H. transiens*	NH 12304	DQ677504	DQ677504	Larsson 2007
*H. tropicum*	CLZhao 17308	OM985727	OM985768	Duan et al. 2023
*H. variolosum*	CBS:734.91	MH862320	MH873992	Vu et al. 2019
*H. variolosum*	CBS:735.91	MH862321	MH873993	Vu et al. 2019
*H. weishanense*	CLZhao 22403	OR141727	OR506181	Yang et al. 2023
*H. yingjiangense*	CLZhao 37815	PV470536	–	Wijesinghe et al. 2025
*H. yingjiangense*	CLZhao 36602	PV470535	–	Wijesinghe et al. 2025
*H. yunnanense*	CLZhao 8845	OM985728	OM985769	Duan et al. 2023
Steccherinum aff. nitidum	FP-105195-Sp	KP135323	KP135227	Floudas and Hibbett 2015
*S. amapaense*	M245	KY977406	KY977405	Hyde et al. 2017
*S. austrosinense*	Dai 17540	MN871755	MN877768	Du et al. 2020
*S. austrosinense*	Dai 17679	MN871756	MN877769	Du et al. 2020
*S. autumnale*	Spirin 2957	JN710549	JN710549	Liu and Dai 2021
*S. bononiae*	AG 1615	PV434851	PV434851	Westphalen et al. 2026
*S. bononiae*	MCW 547/17	PV434850	PV434850	Westphalen et al. 2026
*S. bononiae*	MCW 557/17	PV434854	PV434854	Westphalen et al. 2026
*S. bononiae*	MCW 726/22	PV434852	PV434852	Westphalen et al. 2026
*S. bononiae*	MV446	PV434855	–	Westphalen et al. 2026
*S. bononiae*	NR71	PV434853	PV434853	Westphalen et al. 2026
*S. bourdotii*	HR99893	MT849311	–	Westphalen et al. 2021
*S. bourdotii*	MT 10/19	MT849312	–	Westphalen et al. 2021
*S. bourdotii*	Saarenoksa 10195	JN710584	JN710584	Miettinen et al. 2012
*S. ciliolatum*	Ryvarden 47033	JN710585	JN710585	Miettinen et al. 2012
*S. collabens*	KHL 11848	JN710552	JN710552	Liu and Dai 2021
*S. elegantissimum*	MCW 633/18	PV434856	PV434856	Westphalen et al. 2026
*S. elegantissimum*	MCW 720/21	PV434857	PV434857	Westphalen et al. 2026
*S. elegantissimum*	MCW 721/21	PV434858	–	Westphalen et al. 2026
*S. farinaceum*	CLZhao 37844	PV661644	PV661646	Zhang et al. 2025
*S. farinaceum*	CLZhao 39423	PV661645	PV661647	Zhang et al. 2025
*S. filiferum*	BLS M-5230	OP279612	–	Yurchenko et al. 2023
*S. fimbriatellum*	Miettinen 2091	JN710555	JN710555	Miettinen et al. 2012
*S. fissurutum*	CLZhao 21803	OP799385	OP799397	Dong et al. 2023
*S. fissurutum*	CLZhao 21841	OP799388	OP799400	Dong et al. 2023
*S. formosanum*	Dai 19345	MN871759	MN877772	Du et al. 2020
*S. formosanum*	TFRI 652	EU232184	EU232268	Chou et al. (unpub.)
*S. fragile*	Dai 19972	MW364629	MW364627	Liu and Dai 2021
*S. fragile*	Dai 20479	MW364628	MW364626	Liu and Dai 2021
*S. fragrans*	MG505	PP838792	PP838793	Crous et al. 2024
*S. hirsutum*	CLZhao 4222	MW290040	MW290054	Dong et al. 2022
*S. hirsutum*	CLZhao 4523	MW290041	MW290055	Dong et al. 2022
*S. incrustans*	Dai 19442	ON182084	ON182087	Liu et al. 2023a
*S. juniperi*	Dai 23931	OP956077	OP956031	Liu et al. 2023a
*S. lacerum*	Niemelä 8246	JN710557	JN710557	Miettinen et al. 2012
*S. laeticolor*	Fp-102480-Sp	KY948823	KY948868.1	Justo et al. 2017
*S. larssonii*	MCW 593/17	MT849306	MT849306	Westphalen et al. 2021
*S. larssonii*	MCW 594/17	MT849307	MT849307	Westphalen et al. 2021
*S. laxum*	KHL 12268	JN710577	JN710577	Miettinen et al. 2012
*S. lincangense*	CLZhao 24988	OR096196	OR461455	Dong et al. 2024
*S. longiaculeiferum*	CLZhao 26243	OR096195	–	Dong et al. 2024
*S. longiaculeiferum*	CLZhao 26290	OR167202	–	Dong et al. 2024
*S. meridionalis*	MR 10466	KY174994	KY174994	Westphalen et al. 2018
*S. meridionalis*	MR 284	KY174992	KY174992	Westphalen et al. 2018
*S. molle*	MCW 568/17	PV434863	PV434863	Westphalen et al. 2026
*S. molle*	MCW 641/18	PV434864	PV434864	Westphalen et al. 2026
*S. molle*	MCW 661/18	PV434865	PV434865	Westphalen et al. 2026
*S. molle*	MCW 687/19	PV434866	PV434866	Westphalen et al. 2026
*S. molle*	MCW 734/22	PV434867	–	Westphalen et al. 2026
*S. molle*	MCW 739/23	PV434868	–	Westphalen et al. 2026
*S. molle*	MV450	PV434869	–	Westphalen et al. 2026
*S. nandinae*	Dai 21107	MN833677	MN833679	Du et al. 2020
*S. neonitidum*	MCW 371/12	KY174990	KY174990	Westphalen et al. 2018
*S. neonitidum*	RP 79	KY174991	KY174991	Westphalen et al. 2018
*S. nitidum*	KHL 11903	JN710560	JN710560	Westphalen et al. 2018
*S. nitidum*	MT 33/12	KY174989	KY174989	Westphalen et al. 2018
*S. ochraceum*	2060	JN710589	JN710589	Liu and Dai 2021
*S. ochraceum*	KHL11902	JN710590	JN710590	Westphalen et al. 2021
*S. perparvulum*	524/17	PV434837	PV434837	Westphalen et al. 2026
*S. perparvulum*	543/17	PV434839	PV434839	Westphalen et al. 2026
*S. perparvulum*	592/17	PV434847	PV434847	Westphalen et al. 2026
*S. perparvulum*	659/18	PV434845	PV434845	Westphalen et al. 2026
*S. perparvulum*	692/19	PV434838	PV434838	Westphalen et al. 2026
*S. perparvulum*	710/20	PV434841	–	Westphalen et al. 2026
*S. perparvulum*	742/23	PV434846	–	Westphalen et al. 2026
*S. perparvulum*	744/23	PV434843	–	Westphalen et al. 2026
*S. perparvulum*	MV728	PV434840	–	Westphalen et al. 2026
*S. perparvulum*	MV815	PV434844	PV434844	Westphalen et al. 2026
*S. perparvulum*	NR186	PV434842	–	Westphalen et al. 2026
*S. polycystidiferum*	MCW 419/12	KY174995	KY174995	Westphalen et al. 2018
*S. polycystidiferum*	RP 140	KY174996	KY174996	Westphalen et al. 2018
*S. pseudozilingianum*	Kulju 1004	JN710561	JN710561	Miettinen et al. 2012
*S. pudorinum*	KR-M-0093535	OR381577	—	Popa et al. 2024
*S. puerense*	CLZhao 3122	MW682341	—	Wu et al. 2021a
*S. puerense*	CLZhao 3644	MW682342	MW682338	Wu et al. 2021a
*S. puerense*	Miettinen 13705	JN710592	JN710592	Miettinen et al. 2012
*S. resinaceum*	MCW 540/17	PV434870	PV434870	Westphalen et al. 2026
*S. resinaceum*	MCW 551/17	PV434871	PV434871	Westphalen et al. 2026
*S. resinaceum*	MCW 665/19	PV434872	PV434872	Westphalen et al. 2026
*S. resinaceum*	MCW 679/19	PV434873	PV434873	Westphalen et al. 2026
*S. robustius*	G1195	JN710591	JN710591	Cao et al. 2021
*S. rubigimaculatum*	CLZhao 10638	MW682344	MW682340	Wu et al. 2021a
*S. rubigimaculatum*	CLZhao 4069	MW682343	MW682339	Wu et al. 2021a
***S. shangzhouense***	**Cui 24842**	**PZ458972**	**PZ466730**	**This study**
***S. shangzhouense***	**Cui 24843**	**PZ458973**	**PZ466731**	**This study**
***S. shangzhouense***	**Cui 24845**	**PZ458974**	**PZ466732**	**This study**
*S.* sp.	FD-26	KP135322	KP135289	Floudas and Hibbett 2015
*S.* sp. 2	Miettinen 9300	JN710593	JN710593	Miettinen et al. 2012
*S.* sp. 3	Miettinen 14391	JN710594	JN710594	Miettinen et al. 2012
*S.* sp. 4	Miettinen 13755	JN710596	JN710596	Miettinen et al. 2012
*S. straminellum*	KHL 13849	JN710597	JN710597	Miettinen et al. 2012
*S. subcollabens*	Dai 19344	MN871758	MN877771	Liu and Dai 2021
*S. subcollabens*	Dai 19345	MN871759	MN877772	Liu and Dai 2021
*S. subochraceum*	730/22	PV434859	–	Westphalen et al. 2026
*S. subochraceum*	746/23	PV434861	PV434861	Westphalen et al. 2026
*S. subochraceum*	748/23	PV434860	PV434860	Westphalen et al. 2026
*S. subochraceum*	761/24	PV434862	PV434862	Westphalen et al. 2026
*S. subtropicum*	CLZhao 11059	OP799390	OP799377	Dong et al. 2023
*S. subtropicum*	CLZhao 16901	OP799391	–	Dong et al. 2023
*S. tenue*	5356	JN710598	—	Miettinen et al. 2012
*S. tenue*	KHL 12316	JN710598	JN710598	Miettinen et al. 2012
*S. tenuispinum*	LE231603	KM411452	KM411469	Zmitrovich and Kovalenko 2016
*S. tenuispinum*	Miettinen 8065	JN710599	JN710599	Miettinen et al. 2012
*S. tenuispinum*	Spirin 2116	JN710600	JN710600	Miettinen et al. 2012
*S. undigerum*	MCW 426/13	KY174986	KY174986	Westphalen et al. 2018
*S. undigerum*	MCW 436/13	KY174988	KY174988	Westphalen et al. 2018
*S. undigerum*	MCW 472/13	KY174987	KY174987	Westphalen et al. 2018
*S. undulatum*	MCW 743/23	PV434848	PV434848	Westphalen et al. 2026
*S. undulatum*	MCW 760/24	PV434849	PV434849	Westphalen et al. 2026
*S. weishanense*	CLZhao 24911	OR096207	OR461456	Dong et al. 2024
*S. wumengshanense*	CLZhao 23586	OR658995	OR999392	Wang et al. 2024
*S. xanthum*	CLZhao 5030	MW204588	MW204577	Wu et al. 2021b
*S. xanthum*	CLZhao 5032	MW204589	MW204578	Wu et al. 2021b
*S. yunnanense*	CLZhao 1445	MW290042	MW290056	Dong et al. 2022
*S. yunnanense*	CLZhao 2822	MW290043	MW290057	Dong et al. 2022

### Phylogenetic analysis

Phylogenetic analyses were conducted separately for *Hymenochaete*, *Hyphoderma* and *Steccherinum* based on the combined ITS+nLSU datasets. Reference sequences were retrieved from GenBank (Table 1). In the phylogenetic analyses, *Hydnoporia olivacea* (Schwein.) Teixeira was selected as the outgroup for *Hymenochaete* (Miettinen et al. 2019), *Diplomitoporus crustulinus* (Bres.) Domański was selected as the outgroup for *Hyphoderma* (Justo et al. 2017), and *Cabalodontia delicata* Westph. & Motato-Vásq. was selected as the outgroup for *Steccherinum* (Westphalen et al. 2021).

Sequence alignments were generated using MAFFT v.7 (Katoh et al. 2019) and manually adjusted in BioEdit v.7.0.9 (Hall 1999). Gaps were treated as missing data. The ITS and nLSU alignments were concatenated using Mesquite v.3.2 (Maddison and Maddison 2017).

Maximum Likelihood (ML) analysis was performed using RAxML-HPC2 v.8.2.3 (Stamatakis 2014). The best-scoring ML tree was searched under the GTRGAMMA model, and branch support was assessed with 1000 rapid bootstrap replicates. Bayesian Inference (BI) was performed using MrBayes v.3.2.6 (Ronquist et al. 2012). The substitution model was set to GTR + I + G, with six substitution types and gamma-distributed rate variation across sites with a proportion of invariable sites (nst = 6, rates = invgamma). Base frequencies were assigned a Dirichlet prior. Five Markov Chain Monte Carlo (MCMC) chains were run for 50 million generations, with trees sampled every 100 generations. The temperature parameter was set to 0.2, and the analysis was allowed to stop when the average standard deviation of split frequencies fell below 0.01 (Ronquist and Huelsenbeck 2003). The first 25% of sampled trees were discarded as burn-in, and the remaining trees were used to generate a 50% majority-rule consensus tree and calculate Bayesian posterior probabilities (BPP).

Phylogenetic trees were visualized using FigTree v.1.4.4 (Rambaut 2018). The ML topology is presented, with ML bootstrap values and Bayesian posterior probabilities indicated at the nodes. Only ML-BS ≥ 75% and BPP ≥ 0.90 are shown. Nodes with ML-BS ≥ 75% and BPP ≥ 0.95 were considered well supported.

## Results

### Phylogenetic analyses

To clarify the phylogenetic positions of the newly collected specimens, three independent phylogenetic analyses were conducted for *Hymenochaete*, *Hyphoderma* and *Steccherinum* based on combined ITS+nLSU datasets.

**Figure 1.**
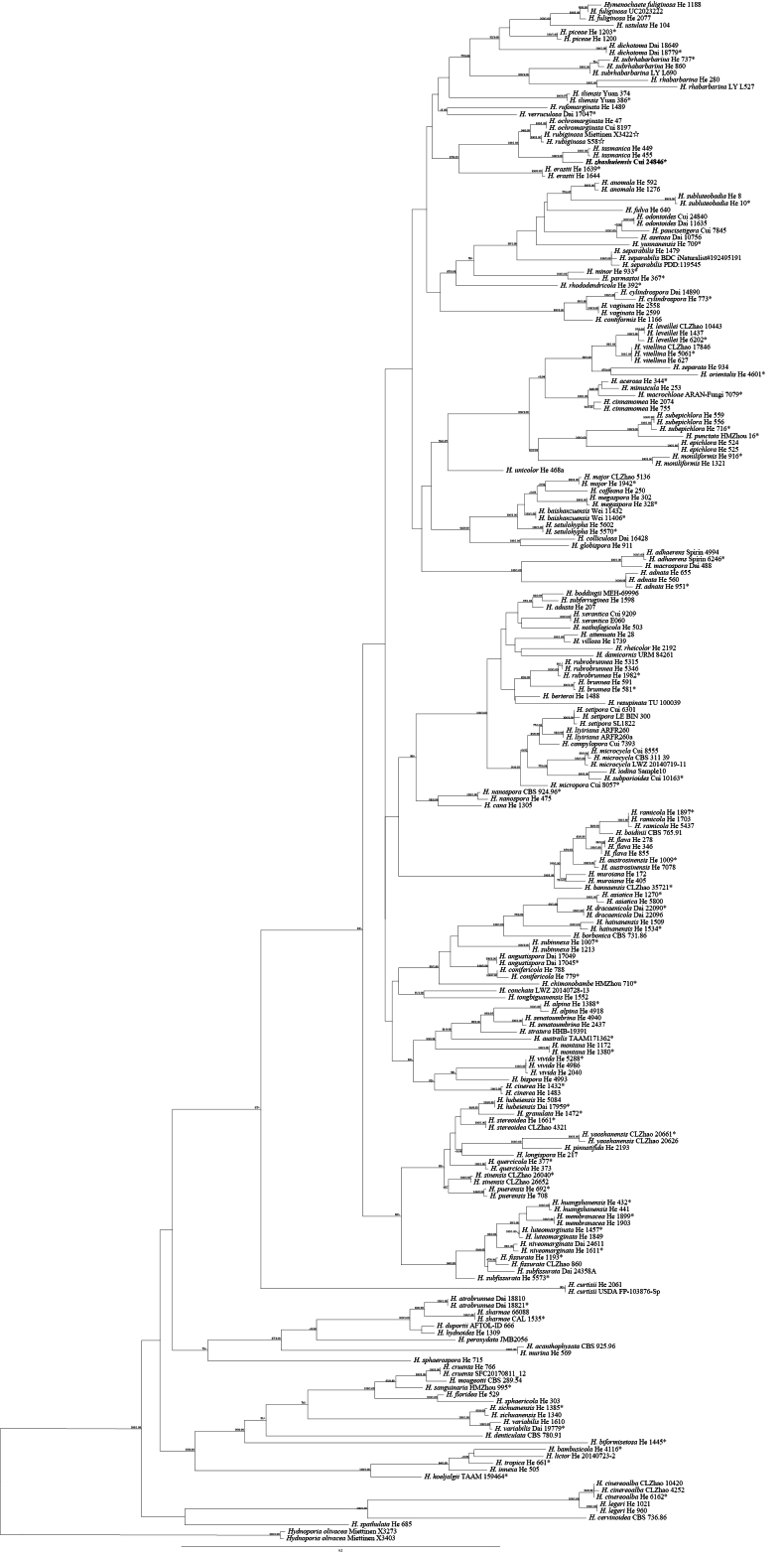
Maximum Likelihood tree illustrating the phylogenetic relationships of *Hymenochaete* inferred from the combined ITS+nLSU dataset. *Hydnoporia olivacea* was used as the outgroup. Branches are labelled with maximum likelihood bootstrap values and Bayesian posterior probabilities. Only maximum likelihood bootstrap values ≥ 75% and Bayesian posterior probabilities ≥ 0.90 are shown. The new taxon is shown in bold. The type species is indicated by white five-pointed star, and type specimens are marked by *.

The combined ITS+nLSU dataset for *Hymenochaete* comprised sequences from 225 fungal samples representing 139 species of *Hymenochaete* and the outgroup species *Hydnoporia olivacea*. The aligned matrix was 2622 characters in length, including 1115 constant characters, 750 parsimony-uninformative variable characters and 757 parsimony-informative characters. Phylogeny results indicated that specimen Cui 24846 formed a distinct lineage (100% ML, 1.00 BPP; Fig. 1), and exhibited a close phylogenetic affinity to *Hymenochaete tasmanica* Massee (Fig. 1).

**Figure 2.**
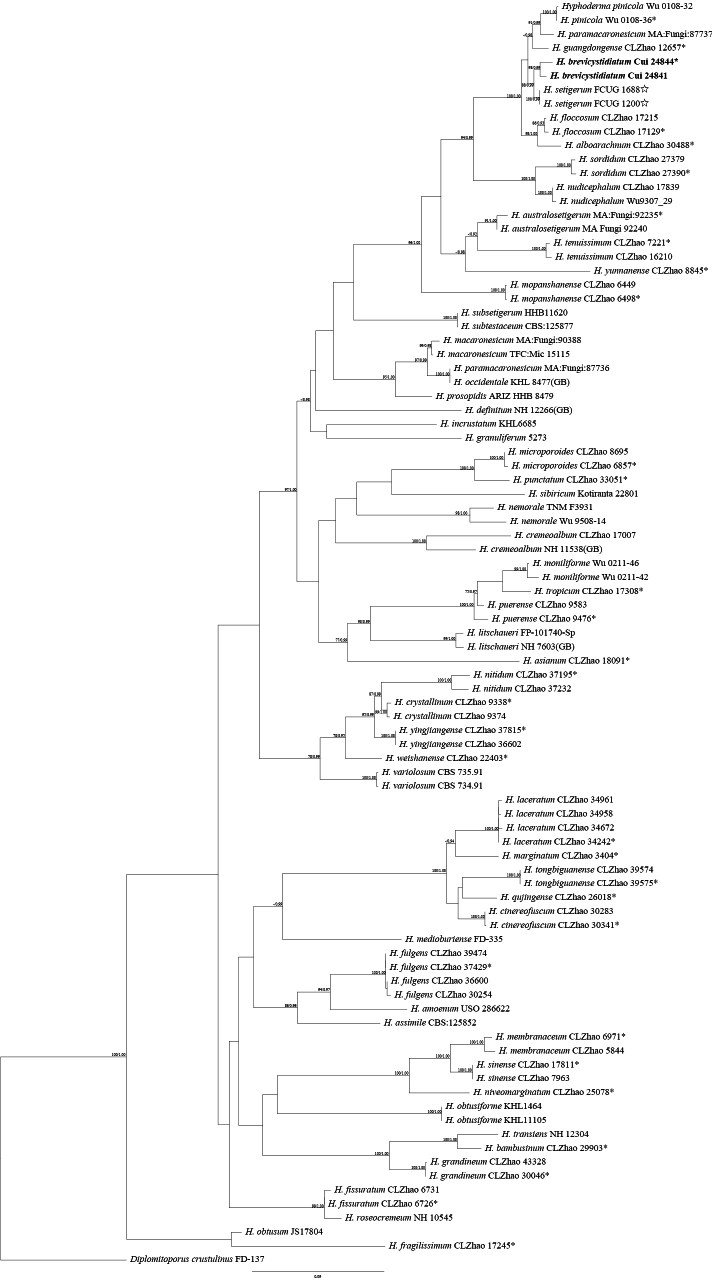
Maximum Likelihood tree illustrating the phylogenetic relationships of *Hyphoderma* inferred from the combined ITS+nLSU dataset. *Diplomitoporus crustulinus* was used as the outgroup. Branches are labelled with maximum likelihood bootstrap values and Bayesian posterior probabilities. Only maximum likelihood bootstrap values ≥ 75% and Bayesian posterior probabilities ≥ 0.90 are shown. The new taxon is shown in bold. The type species is indicated by white five-pointed star, and type specimens are marked by *.

The combined two-gene dataset (ITS+nLSU) for *Hyphoderma* comprised sequences from 91 fungal samples, representing 56 species of *Hyphoderma* and the outgroup species *Diplomitoporus crustulinus*. The aligned matrix was 2072 characters in length, of which 1356 were constant, 164 were parsimony-uninformative variable characters and 552 were parsimony-informative. Phylogenetic analyses revealed that specimens Cui 24841 and Cui 24844 formed a well-supported independent clade (98% ML, 0.99 BPP; Fig. 2) and showed a close phylogenetic relationship with *Hyphoderma setigerum* (Fig. 2). Independent BLAST searches of the ITS and nLSU sequences of Cui 24841 and Cui 24844 were conducted against the NCBI nucleotide database. After exclusion of the query sequence itself, neither gene region yielded close matches to any validly named *Hyphoderma* species. For ITS sequences, the 20 highest-scoring non-self-hits were assigned to *H. subsetigerum* Sheng H. Wu, with sequence identities ranging from 97.15% to 98.83%. For the nLSU sequences, the closest non-self matches included *H. floccosum* C.L. Zhao & Q.X. Guan, *H. guangdongense* J.Q. Su & C.L. Zhao, *H. nudicephalum* Gilb. & M. Blackw., *H. pinicola* Yurchenko & Sheng H. Wu, *H. setigerum*, *H. sordidum* Yang Yang & C.L. Zhao, *H. subsetigerum* and *H. tenuissimum* C.L. Zhao & Q.X. Guan, with sequence identities ranging from 97.88% to 99.88% (Table 2).

**Table 2.** Independent NCBI BLAST comparisons for ITS and nLSU sequences of *Hyphoderma brevicystidiatum* (vouchers Cui 24841 and Cui 24844).

**Query voucher**	**Marker**	**Query accession**	**Query length (bp)**	**Taxa represented among the top 20 non-self-hits**	**Query cover (%)**	**Perident. (%)**
Cui 24841	ITS	PZ458968.1	626	*Hyphoderma subsetigerum* (20/20)	97–100	97.78–98.53
Cui 24841	nLSU	PZ466726.1	1395	*H. floccosum* (4)	95–98	97.88–99.85
				*H. subsetigerum* (2)		
				*H. nudicephalum* (5)		
				*H. guangdongense* (1)		
				*H. sordidum* (1)		
				*H. setigerum* (4)		
				*H. tenuissimum* (2)		
				*Hyphoderma* sp. (1)		
Cui 24844	ITS	PZ458969.1	635	*H. subsetigerum* (20/20)	94–99	97.15–98.83
Cui 24844	nLSU	PZ466727.1	911	*H. pinicola* (4)	93–99	98.00–99.88
				*H. setigerum* (1)		
				*H. subsetigerum* (4)		
				*H.* sp. (1)		
				*H. floccosum* (4)		
				*H. guangdongense* (1)		
				*H. nudicephalum* (5)		

**Figure 3.**
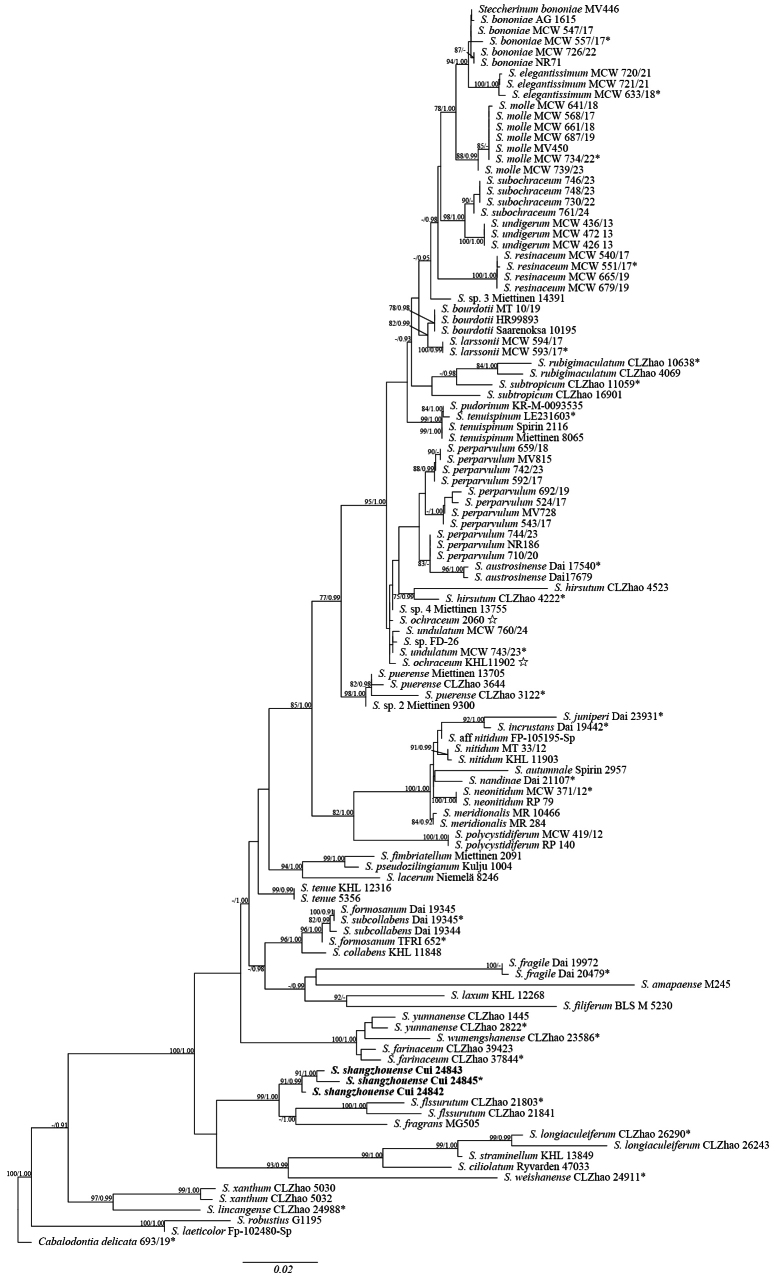
Maximum Likelihood tree illustrating the phylogenetic relationships of *Steccherinum* inferred from the combined ITS+nLSU dataset. *Cabalodontia delicata* was used as the outgroup. Branches are labelled with maximum likelihood bootstrap values and Bayesian posterior probabilities. Only maximum likelihood bootstrap values ≥ 75% and Bayesian posterior probabilities ≥ 0.90 are shown. The new taxon is shown in bold. The type species is indicated by white five-pointed star, and type specimens are marked by *.

The combined ITS+nLSU dataset for *Steccherinum* included sequences from 116 fungal samples representing 56 species of *Steccherinum* and the outgroup species *Cabalodontia delicata*. The aligned matrix was 2016 characters in length, including 1590 constant characters, 125 parsimony-uninformative variable characters and 301 parsimony-informative characters. In the phylogenetic tree, specimens Cui 24842, Cui 24843 and Cui 24845 clustered together and formed an independent lineage within *Steccherinum* (91% ML, 0.99 BPP; Fig. 3), and exhibited a close phylogenetic affinity to *Steccherinum fissurutum* J.H. Dong & C.L. Zhao and *S. fragrans* Ghob.-Nejh. & Langer (Fig. 3).

### Taxonomy

#### Hymenochaete zhashuiensis

Taxon classificationFungiHymenochaetalesHymenochaetaceae

L.L. Wan, L.S. Zhang, Shun Liu & B.K. Cui sp. nov.

138FEDB1-9E05-5DA6-9D80-0CE99C98841E

864506

Figs 4, 5

##### Diagnosis.

*Hymenochaete zhashuiensis* is characterized by resupinate to effused-reflexed basidiomata, a reddish brown to rust-brown, uneven to tuberculate and locally cracked hymenial surface, a monomitic hyphal system with simple-septate, pale yellowish, slightly thick-walled generative hyphae, abundant dark brown, thick-walled hymenial setae (66–89 × 7.8–11 µm), branched dendrohyphidia, and ellipsoid to broadly ellipsoid basidiospores (4–5.6 × 2.8–3.5 µm).

**Figure 4.**
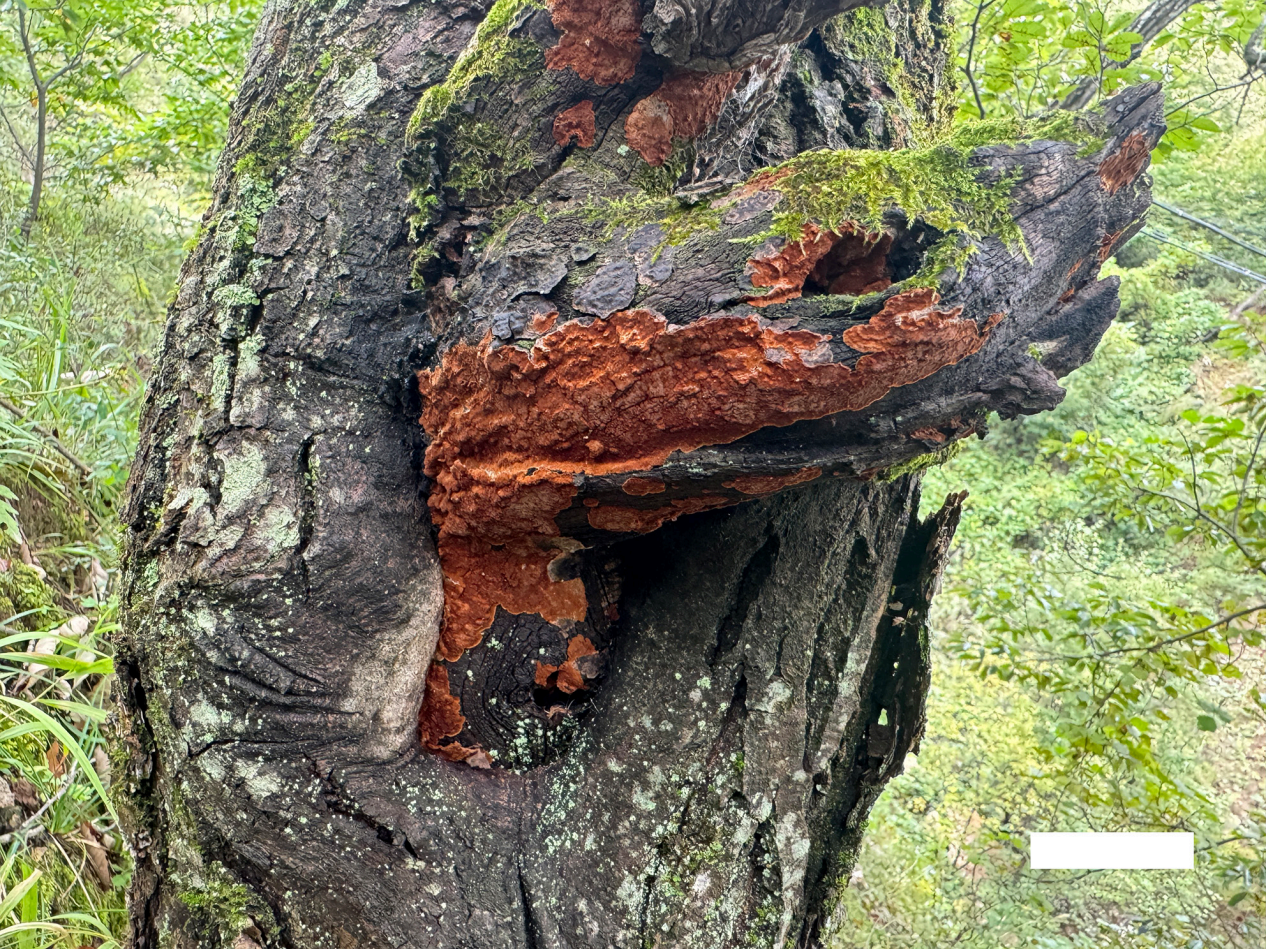
Basidiomata of *Hymenochaete zhashuiensis* (Holotype, Cui 24846). Scale bar: 5 cm.

**Figure 5.**
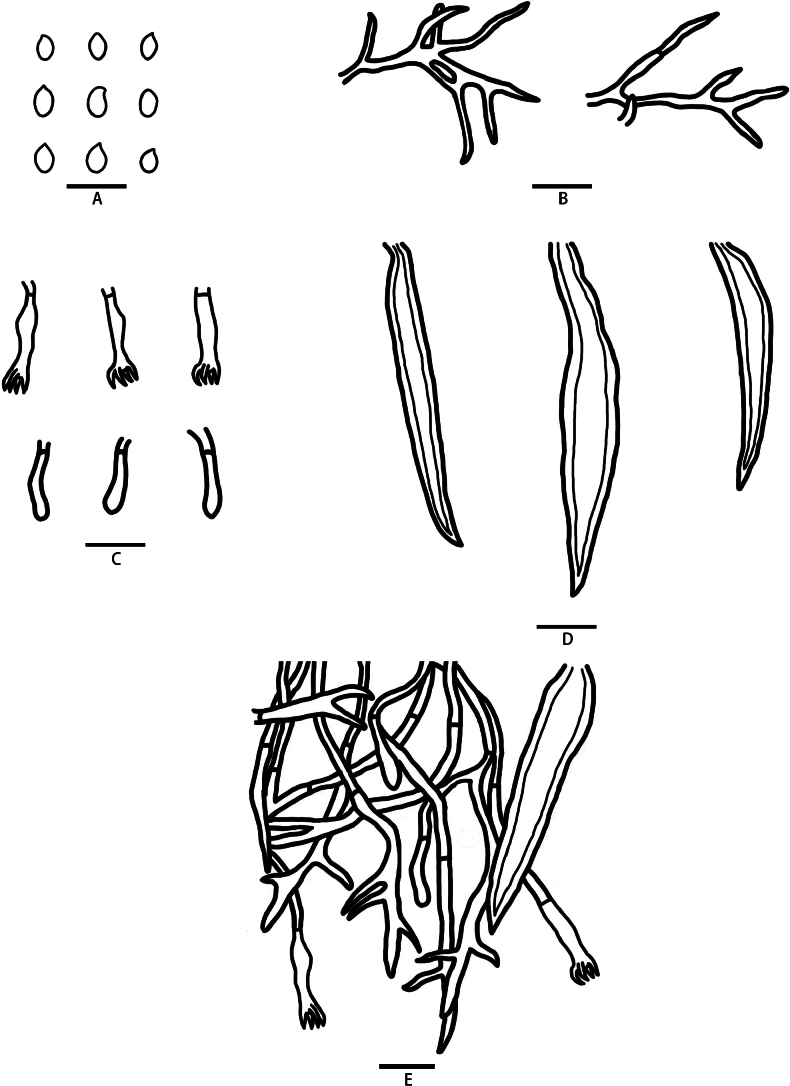
Microscopic structures of *Hymenochaete zhashuiensis* (drawn from Cui 24846). **A**. Basidiospores; **B**. Dendrohyphidia; **C**. Basidia and basidioles; **D**. Setae; **E**. Hymenium. Scale bars: 10 µm (**A–E**).

##### Type.

• China; Shaanxi Province, Zhashui County; 33.807836°N, 108.951155°E; elevation 1184.0 m; on living angiosperm tree; 27 September 2025; Cui 24846 (holotype, BJFC).

##### Etymology.

“*zhashuiensis*” (Lat.): referring to Zhashui County, where the type specimen was collected.

##### Description.

***Fruiting body***. Basidiomata annual, resupinate to effused-reflexed, broadly attached to the substrate, coriaceous to woody when fresh, becoming hard upon drying, 15–20 cm long, 8–12 cm wide, 0.5–3 mm thick. Reflexed part dark brown to blackish brown, rough, uneven, partly covered by mosses and algae in the field. Hymenial surface reddish brown to rust-brown when fresh, becoming dark reddish brown upon drying, uneven, tuberculate to rugose, locally cracked; margin irregular, thinning out, concolorous to slightly paler than the hymenial surface. Tomentum present; cortex absent; hyphal layer present; setal layer well developed.

***Hyphal structure***. Hyphal system monomitic; generative hyphae simple-septate, IKI–, CB–; tissues darkening in KOH.

***Subiculum***. Generative hyphae pale yellowish, slightly thick-walled, frequently branched, interwoven, simple-septate, 1.7–2.3 µm in diam.

***Hymenium***. Hymenial setae abundant, dark brown, thick-walled, fusiform to subulate, straight to slightly curved, with acute apices, projecting from or embedded in the hymenium, 66–89 × 7.8–11.0 µm. Embedded hymenial setae present; true setal hyphae not observed. Cystidia not observed. Dendrohyphidia present, branched, yellowish brown to brown, thin- to slightly thick-walled. Basidia narrowly clavate to subclavate, with four sterigmata and a simple septum at the base, 14.3–17.2 × 2.9–4.1 µm. Basidioles similar to basidia in shape, but smaller.

***Spores***. Basidiospores ellipsoid to broadly ellipsoid, hyaline, thin-walled, smooth, IKI–, CB–, 4–5.6 × 2.8–3.5 µm, L = 4.71 µm, W = 3.19 µm, Q = 1.28–1.84 (n = 30/1).

##### Type of rot.

White rot.

#### Hyphoderma brevicystidiatum

Taxon classificationFungiPolyporalesHyphodermataceae

L.L. Wan, L.S. Zhang, Shun Liu & B.K. Cui sp. nov.

1D1BDD15-6A80-58A4-9688-8F8E229FABA5

864508

Figs 6, 7

##### Diagnosis.

*Hyphoderma brevicystidiatum* is characterized by resupinate, white to cream basidiomata, a smooth to slightly grandinioid hymenial surface, a monomitic hyphal system with clamped generative hyphae, cylindrical to subcylindrical cystidia that are mostly smooth and occasionally bear a few crystalline deposits, subclavate to clavate basidia, and ellipsoid to subcylindrical basidiospores (6.2–10 × 2.7–4.2 µm).

**Figure 6.**
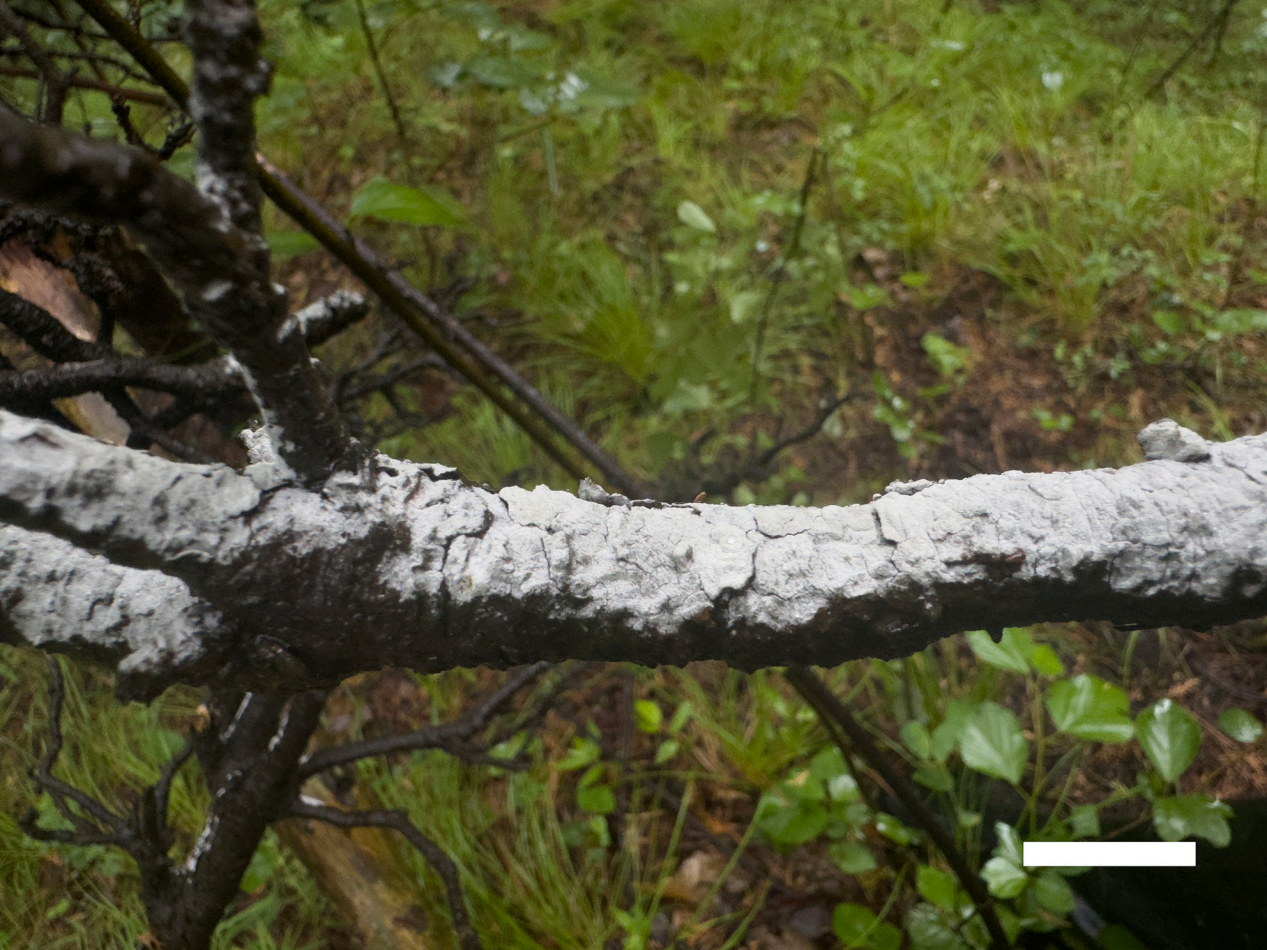
Basidiomata of *Hyphoderma brevicystidiatum* (Holotype, Cui 24844). Scale bar: 2 cm.

**Figure 7.**
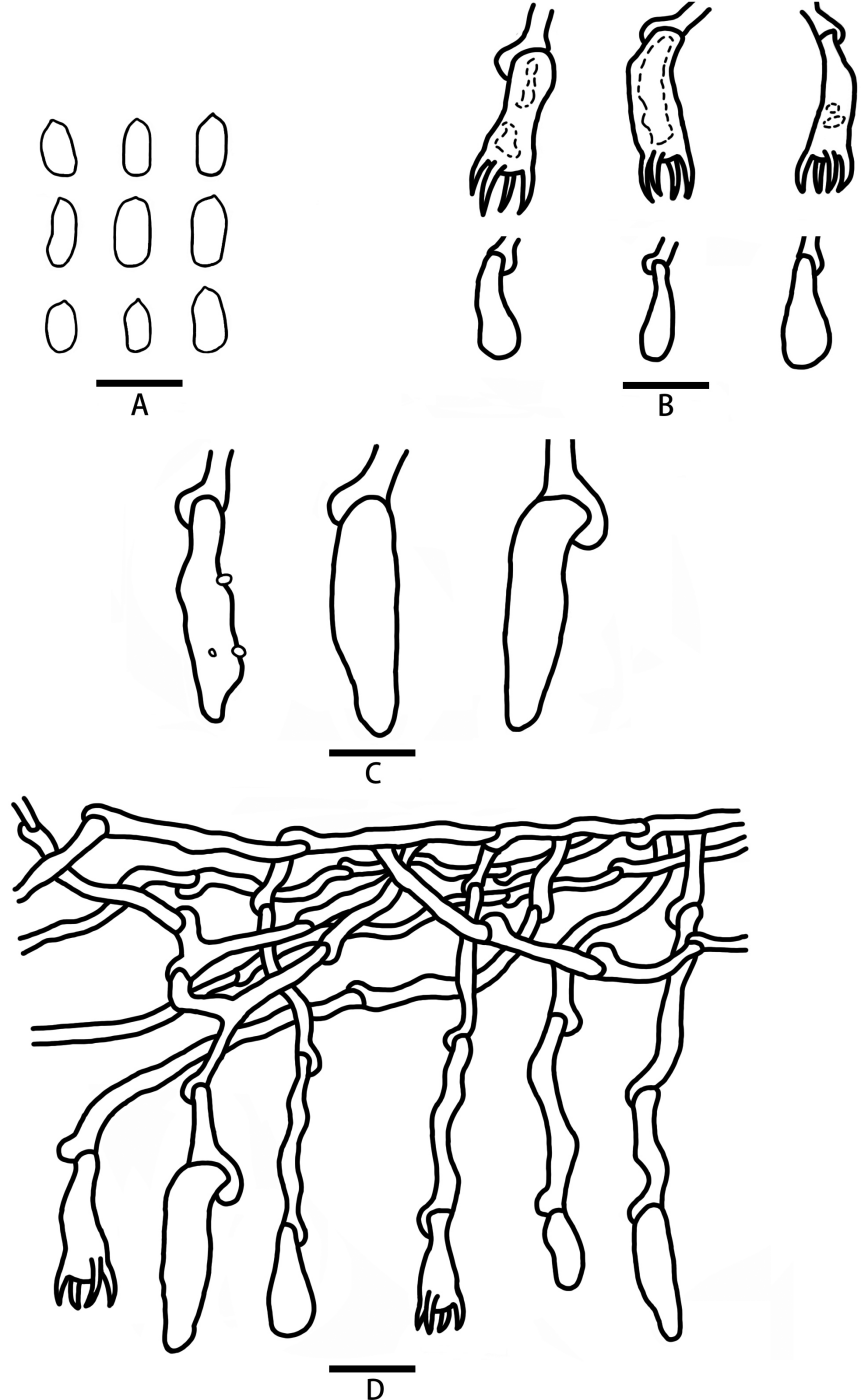
Microscopic structures of *Hyphoderma brevicystidiatum* (Holotype Cui 24844). **A**. Basidiospores; **B**. Basidia and basidioles; **C**. Cystidia; **D**. Part of a vertical section of the hymenium. Scale bars: 10 µm (**A–D**).

##### Type.

• China; Shaanxi Province, Shangzhou District; 33.721046°N, 110.150691°E; elevation 701.6 m; on fallen branch of Pinus; 22 August 2025; Cui 24844 (holotype, BJFC).

##### Etymology.

“*brevicystidiatum*” (Lat.): referring to species having the shorter cystidia.

##### Description.

***Fruiting body***. Basidiomata annual, resupinate, closely adnate to the substrate, membranaceous, soft when fresh, becoming brittle upon drying, 15–23 cm long, 2.5–4 cm wide, 0.3–1 mm thick. Hymenial surface smooth to slightly grandinioid, white to cream when fresh, becoming pale gray upon drying, frequently developing slight cracks.

***Hyphal structure***. Hyphal system monomitic; generative hyphae with clamp connections, IKI–, CB–; tissues unchanged in KOH.

***Subiculum***. Generative hyphae hyaline, thin-walled, frequently branched, interwoven, 1.8–2.5 µm in diameter.

***Hymenium***. Cystidia present, cylindrical to subcylindrical, hyaline, thin- to slightly thick-walled, mostly smooth, occasionally with a few crystalline deposits, 24.2–30.5 × 4.2–7.4 µm. Basidia subclavate to clavate, bearing four sterigmata and a basal clamp connection, 12.8–24.2 × 4.8–6.2 µm. Basidioles similar to basidia in shape, but smaller.

***Spores***. Basidiospores ellipsoid to subcylindrical, hyaline, thin-walled, smooth, IKI–, CB–, (5.2–)6.2–10 (–11.1) × (1.9–)2.7–4.2(–4.4) µm, L = 7.91 µm, W = 3.45 µm, Q = 1.85–2.79 (n = 60/2).

##### Additional specimen (paratype) examined.

• China; Shaanxi Province, Zhashui County; 33.804981°N, 108.931165°E; elevation 1217.7 m; on fallen angiosperm branch; 16 August 2025; Cui 24841 (BJFC).

##### Type of rot.

White rot.

#### Steccherinum shangzhouense

Taxon classificationFungiPolyporalesSteccherinaceae

L.L. Wan, L.S. Zhang, Shun Liu & B.K. Cui sp. nov.

D0019657-FC6F-5F1E-8780-20DF0EF2A36D

864510

Figs 8, 9

##### Diagnosis.

*Steccherinum shangzhouense* is characterized by resupinate and membranaceous basidiomata, white to cream, tuberculate to slightly grandinioid and distinctly cracked hymenial surface, monomitic hyphal system with simple-septate generative hyphae, absence of cystidia and skeletocystidia, clavate basidia, and broadly ellipsoid to oblong ellipsoid basidiospores (5.2–7.9 × 2.5–5 µm).

**Figure 8.**
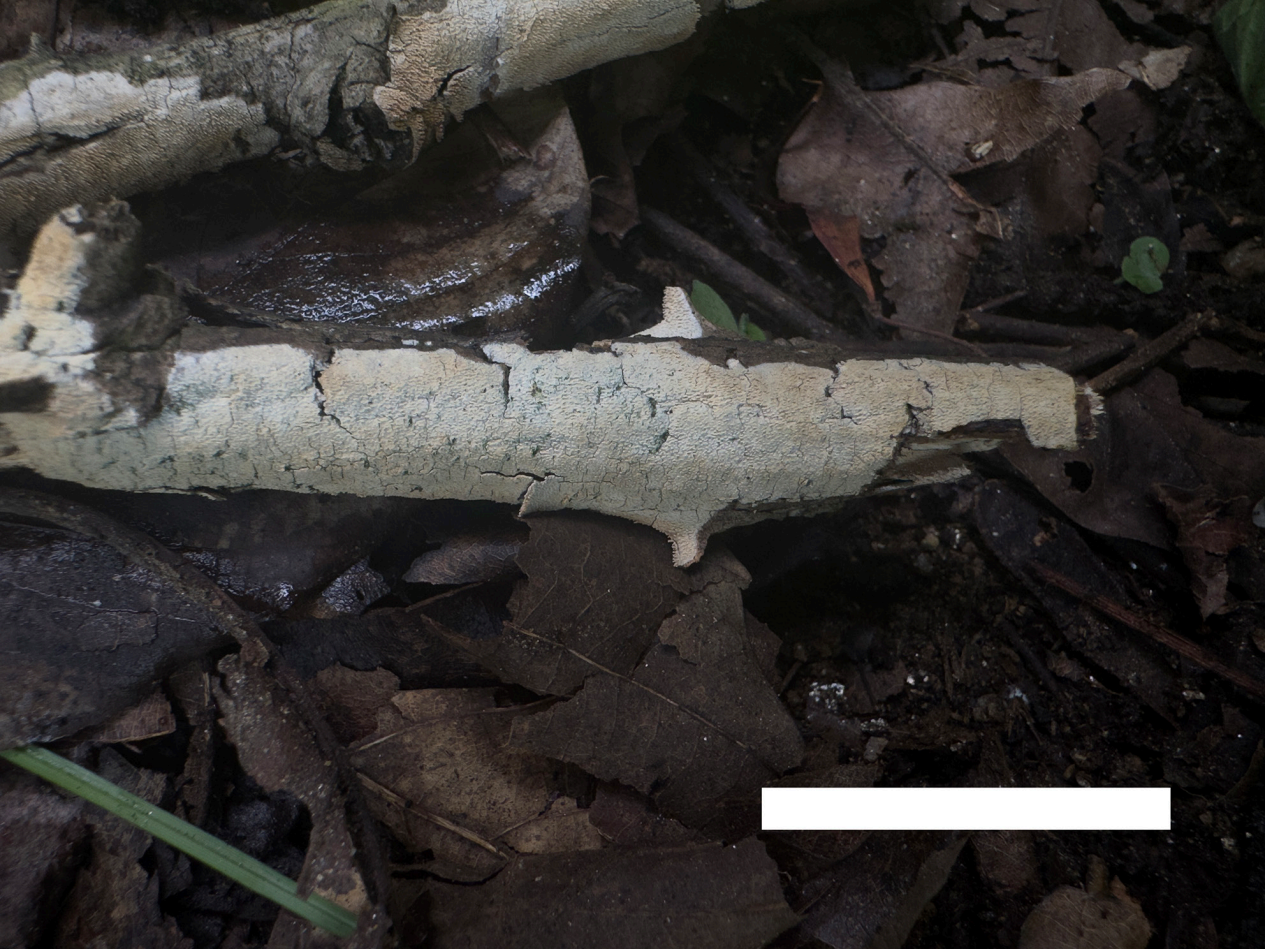
Basidiomata of *Steccherinum shangzhouense* (Holotype, Cui 24845). Scale bar: 2 cm.

**Figure 9.**
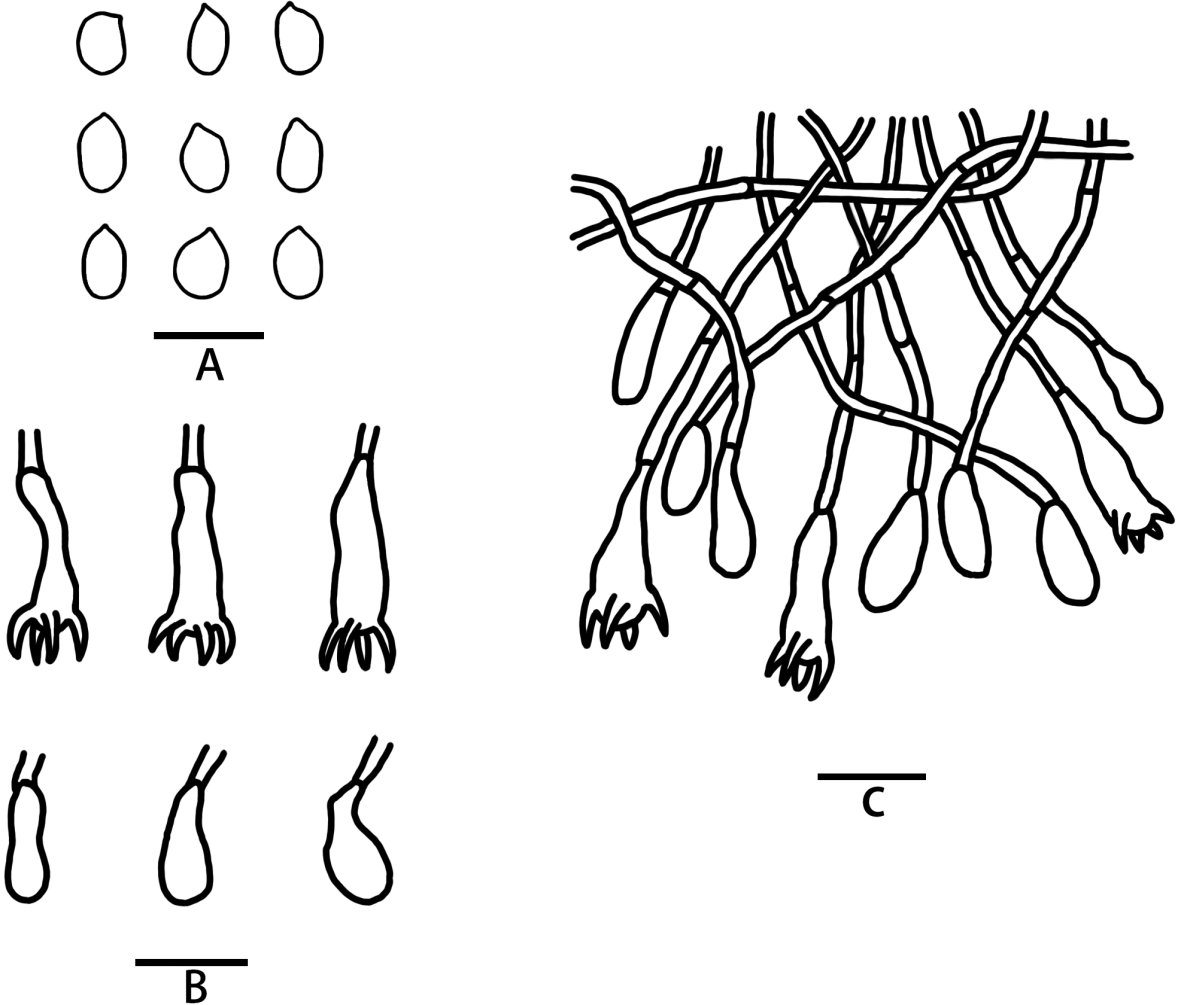
Microscopic structures of *Steccherinum shangzhouense* (drawn from the holotype). **A**. Basidiospores; **B**. Basidia and basidioles; **C**. A section of hymenium. Scale bars: 10 µm (**A–C**).

##### Type.

• China; Shaanxi Province, Shangluo, Shangzhou District; 34.020372°N, 109.983861°E; elevation 852 m; on fallen angiosperm branch; 23 August 2025; Cui 24845 (holotype, BJFC).

##### Etymology.

The epithet “*shangzhouense*” (Lat.): referring to Shangzhou District, Shaanxi Province, where the type specimen was collected.

##### Description.

***Fruiting body***. Basidiomata annual, resupinate, closely adnate to the substrate, membranaceous, soft when fresh, becoming brittle upon drying, white to cream when fresh, turning pale gray to grayish upon drying, frequently developing conspicuous cracks, 13–18 cm long, 1–2 cm wide, and up to 120 µm thick. Hymenial surface tuberculate to slightly grandinioid, composed of irregular granules, white to cream when fresh, becoming pale gray upon drying, distinctly cracked.

***Hyphal structure***. Hyphal system monomitic; generative hyphae simple-septate; hyphae IKI–, CB–; tissues unchanged in KOH.

***Subiculum***. Generative hyphae hyaline, thin-walled, frequently branched, interwoven, 0.8–1.2 µm in diameter.

***Hymenium***. Cystidia and skeletocystidia not observed. Basidia clavate, with four sterigmata and a simple septum at the base, 15.5–20.1 × 3.5–4.8 µm. Basidioles similar to basidia in shape, but smaller.

***Spores***. Basidiospores broadly ellipsoid to oblong ellipsoid, hyaline, thin-walled, smooth, occasionally with one oil drop, IKI–, CB–, (4.3–)5.2–7.9(–8.4) × (1.9–)2.5–5(–5.5) µm, L = 6.44 µm, W = 3.6 µm, Q = 1.51–2.09 (n = 90/3).

##### Additional specimens (paratypes) examined.

• China; Shaanxi Province, Shangzhou District; 33.761057°N, 109.820994°E; elevation 1187 m; on fallen angiosperm trunk; 22 August 2025; Cui 24843 (BJFC). • China; Shaanxi Province, Shangzhou District; 33.760510°N, 109.821477°E; elevation 1206 m; on fallen angiosperm branch; 22 August 2025; Cui 24842 (BJFC).

##### Type of rot.

White rot.

## Discussion

In recent years, numerous new species of wood-inhabiting fungi reported from China have been concentrated in South and Southwest regions (Dai et al. 2004a, 2007, 2009; Yuan and Dai 2008; Zhao et al. 2015; Wu et al. 2016, 2021a, 2021b; Zhang et al. 2023; Zhou et al. 2023), whereas research on the species diversity of corticioid fungi in the Qinling Mountains remains limited. In this study, three new species belonging to the genera *Hymenochaete*, *Hyphoderma* and *Steccherinum* were identified in Zhashui County and Shangzhou District in the eastern Qinling Mountains.

Phylogenetic analysis of *Hymenochaete* revealed that Cui 24846 clustered with *H. tasmanica*, forming a well-supported clade (100% ML, 1.00 BPP; Fig. 1). Morphologically, *H. tasmanica* is similar to *H. zhashuiensis* by sharing a monomitic hyphal system with simple-septate generative hyphae. However, *H. tasmanica* differs by having thinner basidiomata (200–800 µm thick), a smooth to slightly tuberculate and usually uncracked hymenial surface, the presence of a cortex, reddish brown setae, larger basidia (19–25 × 3.5–4.5 µm), and slightly narrower basidiospores (4–5.5 × 2.5–3 µm; He and Li 2012). *Hymenochaete ochromarginata* P.H.B. Talbot and *H. rubiginosa* (Dicks.) Lév. also produce effused-reflexed basidiomata and abundant hymenial setae, but *H. ochromarginata* differs from *H. zhashuiensis* by its yellowish brown to brown pileus with concentrically zonate and sulcate, yellow to ochraceous hymenial margin, shorter hymenial setae (20–56 × 5–10 µm), smaller basidiospores (2–4 × 1.6–2 µm; Vinjusha and Kumar 2024); *H. rubiginosa* differs from *H. zhashuiensis* by its perennial, woody hard basidiomata with distinct cortex, subdimitic hyphal system with sclerefied hyphae, usually uncracked hymenium, and narrower, elongate-ellipsoid basidiospores (3.5–5.5 × 2–3 µm; Corfixen and Parmasto 2017). *Hymenochaete erastii* S.H. He et al. and *H. zhashuiensis* share the presence of a hyphal layer and setal layer and the absence of a cortex, but the former differs by its strictly resupinate, closely adnate basidiomata, grayish violet to clay buff hymenial surface, crystalline masses in the setal layer, smaller setae (30–46 × 3–7 µm), and narrower oblong-ellipsoid to cylindrical basidiospores (4–5 × 1.7–2 µm; Liu et al. 2025).

In the phylogenetic analyses based on combined ITS and nLSU sequences, two specimens of *Hyphoderma brevicystidiatum* (Cui 24841 and Cui 24844) formed an independent lineage with strong nodal support (98% ML, 0.99 BPP; Fig. 2). This species clustered together with *H. alboarachnum* Wen Li & C.L. Zhao, *H. floccosum*, *H. guangdongense*, *H. paramacaronesicum* Tellería et al., *H. pinicola* and *H. setigerum*, constituting a highly supported clade (Fig. 2). Morphologically, all these species possess resupinate basidiomata and a monomitic hyphal system bearing clamp connections. Nevertheless, compared to *Hyphoderma brevicystidiatum*, *H. alboarachnum* differs by its arachnoid hymenial surface, absence of cystidia and cystidioles, cylindrical basidia (23.5–30 × 3.5–4.5 µm), and smaller basidiospores (5–6 × 2–3 µm; Li et al. 2026); *H. floccosum* is distinguished by its farinaceous hymenial surface, two types of cystidia, including thick-walled and abundantly encrusted cystidia (60–161 × 5.5–10 µm) and tubular, thin-walled cystidia (37.5–100 × 4–8.5 µm; Guan and Zhao 2021b); *H. guangdongense* differs by its farinaceous basidiomata, thicker generative hyphae (3.5–6.0 µm), two types of cystidia, including septate cystidia (93–144 × 8–10.4 µm) and tubular cystidia (55–63 × 6.5–10.3 µm), and cylindrical basidiospores (7.4–9 × 3.2–4 µm; Su et al. 2024); *H. paramacaronesicum* is readily distinguished by its yellowish white to pale orange-yellow basidiomata, cylindrical leptocystidia with several constrictions (70–124 × 8–13 µm), larger basidia (40–48 × 6–9 µm), and larger, ellipsoid basidiospores (12–15 × 5.5–7 µm; Martín et al. 2018); *H. pinicola* differs by its chalky white, minutely warted to porulose hymenial surface, larger basidia (25–28 × 5–6.5 µm), longer septocystidia (65–180 µm long), and longer, cylindrical to allantoid basidiospores (13–16 × 4–4.5 µm), as well as its occurrence on dead wood of *Pinus* in Yunnan Province (Yurchenko and Wu 2014a); *H. setigerum* differs in thick-walled and encrusted cystidia (120–180 × 7.5–10 µm), larger basidia (25–40 × 6.5–7 µm), and subcylindrical basidiospores (9.5–11.5 × 3.5–4.5 µm; Nilsson et al. 2003). Furthermore, additional comparisons were made with four members or closely related segregates of the *Hyphoderma setigerum* complex. *Hyphoderma subsetigerum* differs from *H. brevicystidiatum* in having a grandinioid hymenophore with a whitish to ivory-yellow hymenial surface, thick-walled generative hyphae, narrower basidia (20–30 × 4.5–5.5 µm), and smaller basidiospores (6–8 × 2.8–3.2 µm; Wu 1997); *H. bisetigerum* is distinguished by predominantly two-sterigmate basidia and thick-walled, heavily encrusted septocystidia, whereas *H. brevicystidiatum* has four-sterigmate basidia and short, mostly smooth cystidia with only occasional crystalline deposits (Boidin and Gilles 2003); *H. nudicephalum* differs in its farinaceous to odontioid hymenial surface, thick-walled generative hyphae, and conspicuous capitate cystidia with swollen, non-encrusted apices (Gilbertson and Blackwell 1988); *H. tenuissimum* is separated from the new species by its tuberculate to minutely grandinioid, slightly buff hymenial surface, thick-walled generative hyphae, and much longer cylindrical cystidia with 4–12 clamped septa and abundant encrustations (50–220 × 6.5–13 µm), and larger, cylindrical basidiospores (7–10.5 × 3–4.5 µm; Guan et al. 2021).

In the phylogenetic tree of the genus *Steccherinum*, specimens of *S. shangzhouense* (Cui 24842, Cui 24843 and Cui 24845) formed a distinct independent evolutionary lineage, and clustered with *S. fissurutum* and *S. fragrans* to constitute a clade with high statistical support (91% ML, 0.99 BPP; Fig. 3). Nevertheless, these species exhibit conspicuous morphological discrepancies from *S. shangzhouense*. Specifically, *S. fissurutum* differs from *S. shangzhouense* by its subceraceous basidiomata, clamped generative hyphae, presence of numerous strongly encrusted skeletocystidia, and smaller cylindrical basidiospores (4.5–6 × 2.5–3 µm; Dong et al. 2023). *Steccherinum fragrans* differs by its straw-colored basidiomata with a mild sweet smell, raduloid hymenophore with flattened aculei, pseudodimitic hyphal structure, presence of skeletocystidia, and smaller, broadly ellipsoid, cyanophilous basidiospores (3.5–4 × 2.5–2.8 µm; Crous et al. 2024). During wood-inhabiting corticioid fungi investigations in the Qinling Mountains, we also discovered *S. ciliolatum* (Berk. & M.A. Curtis) Gilb. & Budington, *S. longiaculeiferum* J.H. Dong & C.L. Zhao, *S. weishanense* J.H. Dong & C.L. Zhao and *S. straminellum* (Bres.) Melo. Compared with *S. shangzhouense*, *S. ciliolatum* possesses ceraceous to subceraceous basidiomata, well-developed spines up to 1.5 mm long, dimitic hyphal system with clamped generative hyphae, abundant encrusted cystidia (3.5–8 µm wide), longer basidia (18–22 × 4.5–6 µm), and narrower basidiospores (4.5–5.4 × 1.8–2.7 µm; Maas Geesteranus 1974); *S. longiaculeiferum* has coriaceous basidiomata, hydnoid hymenial surface with long aculei, dimitic hyphal system with clamped generative hyphae, and smaller, broadly ellipsoid to ellipsoid basidiospores (4–4.3 × 2.5–3 µm; Dong et al. 2024); *S. weishanense* has coriaceous basidiomata, odontioid hymenial surface with subulate aculei, clamped generative hyphae, two types of cystidia, and smaller ellipsoid basidiospores (4–4.8 × 2.5–3.3 µm; Dong et al. 2024); *S. straminellum* has an odontioid to hydnoid hymenial surface with spines up to 0.5 mm long, fimbriate margin with hyphal strands, dimitic hyphal system with clamped generative hyphae, frequent encrusted cystidia (7–15 µm wide), and narrower basidiospores (3.5–4.5 × 2–2.2 µm; Ryvarden 2024).

Overall, the present study provides additional evidence that the Qinling Mountains harbor a previously underestimated diversity of wood-inhabiting corticioid fungi. The recognition of three new species, *Hymenochaete zhashuiensis*, *Hyphoderma brevicystidiatum* and *Steccherinum shangzhouense*, is supported by both morphological characters and phylogenetic analyses of the combined ITS+nLSU dataset. Continued surveys in the Qinling Mountains are therefore expected to reveal additional undescribed taxa and will contribute to a more comprehensive understanding of the diversity, taxonomy and biogeography of wood-inhabiting fungi in Qinling Mountains, China.

## Supplementary Material

XML Treatment for Hymenochaete zhashuiensis

XML Treatment for Hyphoderma brevicystidiatum

XML Treatment for Steccherinum shangzhouense

## Additional information

### Artificial Intelligence (AI) use

The authors accept full responsibility for the content of the manuscript, including the disclosure of any use of AI.

No AI tools were used in the preparation of this manuscript.

### Author ORCIDs

L.-L. Wan https://orcid.org/0009-0002-9625-0962

L.-S. Zhang https://orcid.org/0009-0002-1832-4516

J.-H. Dong https://orcid.org/0000-0001-8740-0805

L. Zeng https://orcid.org/0009-0004-0124-459X

S. Liu https://orcid.org/0000-0001-9261-4365

B.-K. Cui https://orcid.org/0000-0003-3059-9344

### Data availability

All of the data that support the findings of this study are available in the main text.

## References

[B1] Ariyawansa HA, Hyde KD, Jayasiri SC, Buyck B, Chethana KWT, Dai DQ, Dai YC, Daranagama DA, Jayawardena RS, Lücking R, Ghobad-Nejhad M, Niskanen T, Thambugala KM, Voigt K, Zhao RL, Li GJ, Doilom M, Boonmee S, Yang ZL, Cai Q, Cui YY, Bahkali AH, Chen J, Cui BK, Chen JJ, Dayarathne MC, Dissanayake AJ, Ekanayaka AH, Hashimoto A, Hongsanan S, Jones EBG, Larsson E, Li WJ, Li QR, Liu JK, Luo ZL, Maharachchikumbura SSN, Mapook A, McKenzie EHC, Norphanphoun C, Konta S, Pang KL, Perera RH, Phookamsak R, Phukhamsakda C, Pinruan U, Randrianjohany E, Singtripop C, Tanaka K, Tian CM, Tibpromma S, Abdel-Wahab MA, Wanasinghe DN, Wijayawardene NN, Zhang JF, Zhang H, Abdel-Aziz FA, Wedin M, Westberg M, Ammirati JF, Bulgakov TS, Lima DX, Callaghan TM, Callac P, Chang CH, Coca LF, Dal-Forno M, Dollhofer V, Fliegerová K, Greiner K, Griffith GW, Ho HM, Hofstetter V, Jeewon R, Kang JC, Wen TC, Kirk PM, Kytövuori I, Lawrey JD, Xing J, Li H, Liu ZY, Liu XZ, Liimatainen K, Lumbsch HT, Matsumura M, Moncada B, Nuankaew S, Parnmen S, de Azevedo Santiago ALCM, Sommai S, Song Y, de Souza CAF, de Souza-Motta CM, Su HY, Suetrong S, Wang Y, Wei SF, Wen TC, Yuan HS, Zhou LW, Réblová M, Fournier J, Camporesi E, Luangsa-ard JJ, Tasanathai K, Khonsanit A, Thanakitpipattana D, Somrithipol S, Diederich P, Millanes AM, Common RS, Stadler M, Yan JY, Li XH, Lee HW, Nguyen TTT, Lee HB, Battistin E, Marsico O, Vizzini A, Vila J, Ercole E, Eberhardt U, Simonini G, Wen HA, Chen XH, Miettinen O, Spirin V, Hernawati (2015) Fungal diversity notes 111–252—taxonomic and phylogenetic contributions to fungal taxa. Fungal Diversity 75: 27–274. 10.1007/s13225-015-0346-5

[B2] Baltazar JM, Pildain MB, Gorjón SP, da Silveira RMB, Rajchenberg M (2014) Phylogenetic relationships of *Hydnum peroxydatum* support the synonymy of *Hydnochaete* with *Hymenochaete* (*Hymenochaetaceae*, *Agaricomycetes*). Mycologia 106: 323–327. 10.3852/13-154 24782499

[B3] Bernicchia A, Gorjón SP (2010) Fungi Europaei 12: *Corticiaceae* s.l. Edizioni Candusso, Alassio, 1007 pp.

[B4] Boidin J, Gilles G (2003) *HomobasidiomycètesAphyllophorales* non porés à basides dominantes à 2 (3) stérigmates. Bulletin Trimestriel de la Société Mycologique de France 119: 1–17.

[B5] Boonmee S, Wanasinghe DN, Calabon MS, Huanraluek N, Chandrasiri SKU, Jones GEB, Rossi W, Leonardi M, Singh SK, Rana S, Singh PN, Maurya DK, Lagashetti AC, Choudhary D, Dai YC, Zhao CL, Mu YH, Yuan HS, He SH, Phookamsak R, Jiang HB, Martín MP, Dueñas M, Tellería MT, Kałucka IL, Jagodziński AM, Liimatainen K, Pereira DS, Phillips AJL, Suwannarach N, Kumla J, Khuna S, Lumyong S, Potter TB, Shivas RG, Sparks AH, Vaghefi N, Abdel-Wahab MA, Abdel-Aziz FA, Li GJ, Lin WF, Singh U, Bhatt RP, Lee HB, Nguyen TTT, Kirk PM, Dutta AK, Acharya K, Sarma VV, Niranjan M, Rajeshkumar KC, Ashtekar N, Lad S, Wijesinghe NN, Bhat DJ, Xu RJ, Wijesinghe SN, Shen HW, Luo ZL, Zhang JY, Sysouphanthong P, Thongklang N, Bao DF, Aluthmuhandiram JVS, Abdollahzadeh J, Javadi A, Dovana F, Usman M, Khalid AN, Dissanayake AJ, Telagathoti A, Probst M, Peintner U, Garrido-Benavent I, Bóna L, Merényi Z, Boros L, Zoltán B, Stielow JB, Jiang N, Tian CM, Shams E, Dehghanizadeh F, Pordel A, Javan-Nikkhah M, Denchev TT, Denchev CM, Kemler M, Begerow D, Deng CY, Harrower E, Bozorov T, Kholmuradova T, Gafforov Y, Abdurazakov A, Xu JC, Mortimer PE, Ren GC, Jeewon R, Maharachchikumbura SSN, Phukhamsakda C, Mapook A, Hyde KD (2021) Fungal diversity notes 1387–1511: taxonomic and phylogenetic contributions on genera and species of fungal taxa. Fungal Diversity 111: 1–335. 10.1007/s13225-021-00489-3 PMC8648402 34899100

[B6] Burt EA (1918) The *Thelephoraceae* of North America 10. *Hymenochaete*. Annals of the Missouri Botanical Garden 5: 301–372. 10.2307/2989968

[B7] Cao T, Yu JR, Yuan HS (2021) Multiple-marker phylogeny and morphological evidence reveal two new species in *Steccherinaceae* (*Polyporales*, *Basidiomycota*) from Asia. MycoKeys 78: 169–186. 10.3897/mycokeys.78.57823 PMC8041734 33883969

[B8] Corfixen P, Parmasto E (2017) *Hymenochaete* and *Hymenochaetopsis (Basidiomycota)* in Europe. Karstenia 57: 49–80. 10.29203/ka.2017.483

[B9] Crous PW, Wingfield MJ, Burgess TI, Carnegie AJ, Hardy GESJ, Smith D, Summerell BA, Cano-Lira JF, Guarro J, Houbraken J, Lombard L, Martín MP, Sandoval-Denis M, Alexandrova AV, Barnes CW, Baseia IG, Bezerra JDP, Guarnaccia V, May TW, Hernández-Restrepo M, Stchigel AM, Miller AN, Ordoñez ME, Abreu VP, Accioly T, Agnello C, Agustin Colmán A, Albuquerque CC, Alfredo DS, Alvarado P, Araújo-Magalhães GR, Arauzo S, Atkinson T, Barili A, Barreto RW, Bezerra JL, Cabral TS, Camello Rodríguez F, Cruz RHSF, Daniëls PP, da Silva BDB, de Almeida DAC, de Carvalho Júnior AA, Decock CA, Delgat L, Denman S, Dimitrov RA, Edwards J, Fedosova AG, Ferreira RJ, Firmino AL, Flores JA, García D, Gené J, Giraldo A, Góis JS, Gomes AAM, Gonçalves CM, Gouliamova DE, Groenewald M, Guéorguiev BV, Guevara-Suarez M, Gusmão LFP, Hosaka K, Hubka V, Huhndorf SM, Jadan M, Jurjević Ž, Kraak B, Kučera V, Kumar TKA, Kušan I, Lacerda SR, Lamlertthon S, Lisboa WS, Loizides M, Luangsa-Ard JJ, Lysková P, Mac Cormack WP, Macedo DM, Machado AR, Malysheva EF, Marinho P, Matočec N, Meijer M, Mešić A, Mongkolsamrit S, Moreira KA, Morozova OV, Nair KU, Nakamura N, Noisripoom W, Olariaga I, Oliveira RJV, Paiva LM, Pawar P, Pereira OL, Peterson SW, Prieto M, Rodríguez-Andrade E, Rojo De Blas C, Roy M, Santos ES, Sharma R, Silva GA, Souza-Motta CM, Takeuchi-Kaneko Y, Tanaka C, Thakur A, Smith MT, Tkalčec Z, Valenzuela-Lopez N, van der Kleij P, Verbeken A, Viana MG, Wang XW, Groenewald JZ (2017) Fungal Planet description sheets: 625–715. Persoonia 39: 270–467. 10.3767/persoonia.2017.39.11 PMC5832955 29503478

[B10] Crous PW, Wingfield MJ, Jurjević Ž, Balashov S, Osieck ER, Marin-Felix Y, Luangsa-ard JJ, Mejía LC, Cappelli A, Parra LA, Lucchini G, Chen J, Moreno G, Faraoni M, Zhao RL, Weholt Ø, Borovička J, Jansen GM, Shivas RG, Tan YP, Akulov A, Alfenas AC, Alfenas RF, Altés A, Avchar R, Barreto RW, Catcheside DEA, Chi TY, Esteve-Raventós F, Fryar SC, Hanh LTM, Larsbrink J, Oberlies NH, Olsson L, Pancorbo F, Raja HA, Thanh VN, Thuy NT, Ajithkumar K, Akram W, Alvarado P, Angeletti B, Arumugam E, Atashi Khalilabad A, Bandini D, Baroni TJ, Barreto GG, Boertmann D, Bose T, Castañeda-Ruiz RF, Couceiro A, Cykowska-Marzencka B, Dai YC, Darmostuk V, da Silva SBG, Dearnaley JDW, de Azevedo Santiago ALCM, Declercq B, de Freitas LWS, De la Peña-Lastra S, Delgado G, de Lima CLF, Dhotre D, Dirks AC, Eisvand P, Erhard A, Ferro LO, García D, García-Martín A, Garrido-Benavent I, Gené J, Ghobad-Nejhad M, Gore G, Gunaseelan S, Gusmão LFP, Hammerbacher A, Hernández-Perez AT, Hernández-Restrepo M, Hofmann TA, Hubka V, Jiya N, Kaliyaperumal M, Keerthana KS, Ketabchi M, Kezo K, Knoppersen R, Kolarczyková D, Kumar TKA, Læssøe T, Langer E, Larsson E, Lodge DJ, Lynch MJ, Maciá-Vicente JG, Mahadevakumar S, Mateos A, Mehrabi-Koushki M, Miglio BV, Noor A, Oliveira JA, Pereira OL, Piątek M, Pinto A, Ramírez GH, Raphael B, Rawat G, Renuka M, Reschke K, Ruiz Mateo A, Saar I, Saba M, Safi A, Sánchez RM, Sandoval-Denis M, Savitha AS, Sharma A, Shelke D, Sonawane H, Souza MGAP, Stryjak-Bogacka M, Thines M, Thomas A, Torres-García D, Traba JM, Vauras J, Vermaas M, Villarreal M, Vu D, Whiteside EJ, Zafari D, Starink-Willemse M, Groenewald JZ (2024) Fungal Planet description sheets: 1697–1780. Fungal Systematics and Evolution 14: 325–577. 10.3114/fuse.2024.14.19 PMC11736264 39830292

[B11] Dai YC (2010) *Hymenochaetaceae (Basidiomycota)* in China. Fungal Diversity 45: 131–343. 10.1007/s13225-010-0066-9

[B12] Dai YC, Wei YL, Wang Z (2004a) Wood-inhabiting fungi in southern China 2. Polypores from Sichuan Province. Annales Botanici Fennici 41: 319–329.

[B13] Dai YC, Wei YL, Zhang XQ (2004b) An annotated checklist of non-poroid *Aphyllophorales* in China. Annales Botanici Fennici 41: 233–247.

[B14] Dai YC, Yu CJ, Wang HC (2007) Polypores from eastern Xizang (Tibet), western China. Annales Botanici Fennici 44: 135–145.

[B15] Dai YC, Yuan HS, Wang HC, Yang F, Wei YL (2009) Polypores (*Basidiomycota*) from Qin Mts. in Shaanxi Province, central China. Annales Botanici Fennici 46: 54–61. 10.5735/085.046.0105

[B16] Deng YL, Chen M, Liu LF, Li QZ, Zhang SC, Yuan HS, Zhao CL (2025) Morphological and molecular analyses revealed four new wood-inhabiting fungal species (*Hymenochaetales*, *Basidiomycota*) from Yunnan. MycoKeys 117: 29–66. 10.3897/mycokeys.117.146236 PMC12059579 40351353

[B17] Dong JH, Wu YX, Zhao CL (2022) Two new species of *Steccherinum* (*Polyporales*, *Basidiomycota*) from southern China based on morphology and DNA sequence data. Mycoscience 63: 65–72. 10.47371/mycosci.2022.02.002 PMC10024968 37092008

[B18] Dong JH, Zhang XC, Chen JJ, Zhu ZL, Zhao CL (2023) A phylogenetic and taxonomic study on *Steccherinum* (*Polyporales*, *Basidiomycota*): Focusing on three new *Steccherinum* species from southern China. Frontiers in Cellular and Infection Microbiology 12: 1103579. 10.3389/fcimb.2022.1103579 PMC9930103 36817691

[B19] Dong JH, Li Q, Yuan Q, Luo YX, Zhang XC, Dai YF, Zhou Q, Liu XF, Deng YL, Zhou HM, Muhammad A, Zhao CL (2024) Species diversity, taxonomy, molecular systematics and divergence time of wood-inhabiting fungi in Yunnan-Guizhou Plateau, Asia. Mycosphere 15: 1110–1293. 10.5943/mycosphere/15/1/10

[B20] Donk MA (1957) Notes on resupinate *Hymenomycetes* IV. Fungus 27: 1–29.

[B21] Du P, Wu F, Tian XM (2020) Three new species of *Junghuhnia* (*Polyporales*, *Basidiomycota*) from China. MycoKeys 72: 1–16. 10.3897/mycokeys.72.51872 PMC7442768 32879617

[B22] Du P, Cao T-X, Wu Y-D, Zhou M, Liu ZB (2021) Two new species of *Hymenochaetaceae* on *Dracaena cambodiana* from tropical China. MycoKeys 80: 1–17. 10.3897/mycokeys.80.63997 PMC8116325 34007241

[B23] Duan ZY, Guan QX, Luo KY, Zhao CL (2023) Morphological and molecular identification of three new resupinate species of *Hyphoderma* (*Hyphodermataceae*, *Agaricomycetes*) from East Asia. Phytotaxa 599: 1–19. 10.11646/phytotaxa.599.1.1

[B24] Floudas D, Hibbett DS (2015) Revisiting the taxonomy of *Phanerochaete* (*Polyporales*, *Basidiomycota*) using a four-gene dataset and extensive ITS sampling. Fungal Biology 119: 679–719. 10.1016/j.funbio.2015.04.003 26228559

[B25] Fröhlich-Nowoisky J, Pickersgill DA, Després VR, Pöschl U (2009) High diversity of fungi in air particulate matter. Proceedings of the National Academy of Sciences of the United States of America 106(31): 12814–12819. 10.1073/pnas.0811003106 PMC2722276 19617562

[B26] Gilbertson RL, Blackwell M (1988) Some new or unusual corticioid fungi from the Gulf Coast region. Mycotaxon 33: 375–386. 10.5962/p.416675

[B27] Gray SF (1821) A Natural Arrangement of British Plants, Vol. 1. Baldwin, Cradock, and Joy, London.

[B28] Guan QX, Zhao CL (2021a) Taxonomy and phylogeny of the wood-inhabiting fungal genus *Hyphoderma* with descriptions of three new species from East Asia. Journal of Fungi 7: 308. 10.3390/jof7040308 PMC8072537 33923807

[B29] Guan QX, Zhao CL (2021b) Two new corticioid species: *Hyphoderma sinense* and *H. floccosum* spp. nov. (*Hyphodermataceae*, *Polyporales*) from southern China. Mycosystema 40: 447–461. 10.13346/j.mycosystema.200382

[B30] Guan QX, Li YF, Zhao CL (2021) Morphological and phylogenetic evidence for recognition of two new species of *Hyphoderma (Basidiomycota)* from southern China, with a key to all Chinese *Hyphoderma*. MycoKeys 83: 145–160. 10.3897/mycokeys.83.69909 PMC8476483 34629930

[B31] Hall TA (1999) BioEdit: a user-friendly biological sequence alignment editor and analysis program for Windows 95/98/NT. Nucleic Acids Symposium Series 41: 95–98.

[B32] He SH, Dai YC (2012) Taxonomy and phylogeny of *Hymenochaete* and allied genera of *Hymenochaetaceae (Basidiomycota)* in China. Fungal Diversity 56: 77–93. 10.1007/s13225-012-0174-9

[B33] He SH, Li HJ (2011) Two new species of *Hymenochaete (Hymenochaetales)* from China. Mycotaxon 115: 375–382. 10.5248/115.375

[B34] He SH, Li HJ (2012) *Hymenochaete* in China 1. Three species new to China from Guangdong and Anhui provinces. Mycosystema 31: 50–54. 10.13346/j.mycosystema.2012.01.016

[B35] He SH, Li HJ (2013a) *Hymenochaete* in China. 2. A new species and three new records from Yunnan Province. Mycological Progress 12: 331–339. 10.1007/s11557-012-0838-6

[B36] He SH, Li HJ (2013b) *Hymenochaete* in China. 4. *H. parmastoi* sp. nov. and *H. ustulata* new to China. Mycotaxon 122: 197–205. 10.5248/122.197

[B37] He SH, Li HJ (2014) *Hymenochaete* (*Hymenochaetales*, *Basidiomycota*) in China 7. *H. cana* sp. nov. and *H. denticulata* new to China. Chiang Mai Journal of Science 41(4): 781–788.

[B38] He SH, Liu SL, Li HJ, Chen JJ, Cui BK (2017) Two new species of *Hymenochaete* (*Hymenochaetaceae*, *Basidiomycota*) and *H. colliculosa* new to China from Shanxi Province. Phytotaxa 324: 168–178. 10.11646/phytotaxa.324.2.5

[B39] Hopple Jr JS, Vilgalys R (1999) Phylogenetic relationships in the mushroom genus *Coprinus* and dark-spored allies based on sequence data from the nuclear gene coding for the large ribosomal subunit RNA: divergent domains, outgroups, and monophyly. Molecular Phylogenetics and Evolution 13: 1–19. 10.1006/mpev.1999.0634 10508535

[B40] Hyde KD, Norphanphoun C, Abreu VP, Bazzicalupo A, Chethana KWT, Clericuzio M, Dayarathne MC, Dissanayake AJ, Ekanayaka AH, He MQ, Hongsanan S, Huang SK, Jayasiri SC, Jayawardena RS, Karunarathna A, Konta S, Kušan I, Lee H, Li JF, Lin CG, Liu NG, Lu YZ, Luo ZL, Manawasinghe IS, Mapook A, Perera RH, Phookamsak R, Phukhamsakda C, Siedlecki I, Soares AM, Tennakoon DS, Tian Q, Tibpromma S, Wanasinghe DN, Xiao YP, Yang J, Zeng XY, Abdel-Aziz FA, Li WJ, Senanayake IC, Shang QJ, Daranagama DA, de Silva NI, Thambugala KM, Abdel-Wahab MA, Bahkali AH, Berbee ML, Boonmee S, Bhat DJ, Bulgakov TS, Buyck B, Camporesi E, Castañeda-Ruiz RF, Chomnunti P, Doilom M, Dovana F, Gibertoni TB, Jadan M, Jeewon R, Jones EBG, Kang JC, Karunarathna SC, Lim YW, Liu JK, Liu ZY, Plautz Jr HL, Lumyong S, Maharachchikumbura SSN, Matočec N, McKenzie EHC, Mešić A, Miller D, Pawłowska J, Pereira OL, Promputtha I, Romero AI, Ryvarden L, Su HY, Suetrong S, Tkalčec Z, Vizzini A, Wen TC, Wisitrassameewong K, Wrzosek M, Xu JC, Zhao Q, Zhao RL, Mortimer PE (2017) Fungal diversity notes 603–708: taxonomic and phylogenetic notes on genera and species. Fungal Diversity 87: 1–235. 10.1007/s13225-017-0391-3

[B41] Justo A, Miettinen O, Floudas D, Ortiz-Santana B, Sjökvist E, Lindner D, Nakasone K, Niemelä T, Larsson KH, Ryvarden L, Hibbett DS (2017) A revised family-level classification of the *Polyporales (Basidiomycota)*. Fungal Biology 121: 798–824. 10.1016/j.funbio.2017.05.010 28800851

[B42] Katoh K, Rozewicki J, Yamada KD (2019) MAFFT online service: Multiple sequence alignment, interactive sequence choice and visualization. Briefings in Bioinformatics 20: 1160–1166. 10.1093/bib/bbx108 PMC6781576 28968734

[B43] Kobayashi T, Oguro M, Akiba M, Taki H, Kitajima H, Ishihara H (2020) Mushroom yield of cultivated shiitake (*Lentinula edodes*) and fungal communities in logs. Journal of Forest Research 25(4): 269–275. 10.1080/13416979.2020.1759886

[B44] Kornerup A, Wanscher JH (1967) Methuen Handbook of Colour. Methuen, London.

[B45] Larsson KH (2007) Re-thinking the classification of corticioid fungi. Mycological Research 111: 1040–1063. 10.1016/j.mycres.2007.08.001 17981020

[B46] Léger JC (1998) Le genre *Hymenochaete* Léveillé. Bibliotheca Mycologica 171: 1–319.

[B47] Léveillé JH (1846) Descriptions de champignons de l’herbier du Muséum de Paris. Annales des Sciences Naturelles, Botanique III 5: 111–167.

[B48] Li W, Wang XY, Chen DX, Zhang SC, Li YC, Zhao CL, Zhan ZY (2025) Molecular phylogeny and morphology reveal a new wood-inhabiting fungus *Hyphoderma cinereofuscum* (*Polyporales*, *Basidiomycota*), from southwest China. Phytotaxa 683: 106–118. 10.11646/phytotaxa.683.2.2

[B49] Li W, Wang H, Wang X, Liao X, Wang K, Subodini Nuwanthika WA, Zhao C (2026) Phylogeny, divergence time and historical biogeography of *Hyphoderma* (*Hyphodermataceae*, *Polyporales*): Introducing five new species from China. MycoKeys 129: 43–80. 10.3897/mycokeys.129.183371 PMC12946824 41766951

[B50] Li YC, Chen ML, Xiao WY, Zhang JZ, Zhao CL (2024) Molecular phylogeny and morphology reveal a new *Hymenochaete* species (*Hymenochaetaceae*, *Basidiomycota*) from China. Phytotaxa 664(3): 159–171. 10.11646/phytotaxa.664.3.1

[B51] Liu S, Shen LL, Xu TM, Song CG, Gao N, Wu DM, Sun YF, Cui BK (2023a) Global diversity, molecular phylogeny and divergence times of the brown-rot fungi within the *Polyporales*. Mycosphere 14: 1564–1664. 10.5943/mycosphere/14/1/18

[B52] Liu ZB, Dai YC (2021) *Steccherinum fragile* sp. nov. and *S. subcollabens* comb. nov. (*Steccherinaceae*, *Polyporales*), evidenced by morphological characters and phylogenetic analysis. Phytotaxa 483: 106–116. 10.11646/phytotaxa.483.2.3

[B53] Liu ZB, Zhou M, Zhang QY, Si J (2023b) A contribution to the genus *Steccherinum* (*Steccherinaceae*, *Polyporales*): Introducing two new species and two new combinations of the genus. Frontiers in Microbiology 14: 1166267. 10.3389/fmicb.2023.1166267 PMC10073545 37032844

[B54] Liu ZB, Yuan Y, Dai YC, Liu HG, Vlasák J, Zeng GY, He SH, Wu F (2025) Global diversity and systematics of *Hymenochaetaceae* with non-poroid hymenophore. Fungal Diversity 131: 1–97. 10.1007/s13225-025-00552-3

[B55] Ma X, Huang RX, Zhang Y, Zhao CL (2021) *Hyphoderma fissuratum* and *H. mopanshanense* spp. nov. (*Polyporales*) from southern China. Mycoscience 62: 36–41. 10.47371/mycosci.2020.08.004 PMC9157751 37090020

[B56] Maas Geesteranus RA (1974) Studies in the genera *Irpex* and *Steccherinum*. Persoonia 7: 443–581.

[B57] Maddison WP, Maddison DR (2017) Mesquite: a modular system for evolutionary analysis. Version 3.2. http://mesquiteproject.org

[B58] Martín MP, Zhang LF, Fernández-López J, Dueñas M, Rodríguez-Armas JL, Beltrán-Tejera E, Tellería MT (2018) *Hyphoderma paramacaronesicum* sp. nov. (*Meruliaceae*, *Polyporales*, *Basidiomycota*), a cryptic lineage to *H. macaronesicum*. Fungal Systematics and Evolution 2: 57–68. 10.3114/fuse.2018.02.05 PMC7225581 32467888

[B59] Matheny PB, Curtis JM, Hofstetter V, Aime MC, Moncalvo JM, Ge Z-W, Yang ZL, Slot JC, Ammirati JF, Baroni TJ, Bougher NL, Hughes KW, Lodge DJ, Kerrigan RW, Seidl MT, Aanen DK, DeNitis M, Daniele GM, Desjardin DE, Kropp BR, Norvell LL, Parker A, Vellinga EC, Vilgalys R, Hibbett DS (2006) Major clades of *Agaricales*: A multilocus phylogenetic overview. Mycologia 98(6): 982–995. 10.3852/mycologia.98.6.982 17486974

[B60] Miettinen O, Larsson E, Sjökvist E, Larsson KH (2012) Comprehensive taxon sampling reveals unaccounted diversity and morphological plasticity in a group of dimitic polypores (*Polyporales*, *Basidiomycota*). Cladistics 28: 251–270. 10.1111/j.1096-0031.2011.00380.x 34872189

[B61] Miettinen O, Larsson KH, Spirin V (2019) *Hydnoporia*, an older name for *Pseudochaete* and *Hymenochaetopsis*, and typification of the genus *Hymenochaete* (*Hymenochaetales*, *Basidiomycota*). Fungal Systematics and Evolution 4: 77–96. 10.3114/fuse.2019.04.07 PMC7241676 32467908

[B62] Nie T, Tian Y, Liu SL, Yang J, He SH (2017) Species of *Hymenochaete* (*Hymenochaetales*, *Basidiomycota*) on bamboos from East Asia, with descriptions of two new species. MycoKeys 20: 51–65. 10.3897/mycokeys.20.11754

[B63] Nilsson RH, Hallenberg N, Nordén B, Maekawa N, Wu SH (2003) Phylogeography of *Hyphoderma setigerum* (*Basidiomycota*) in the northern hemisphere. Mycological Research 107: 645–652. 10.1017/S0953756203007925 12951791

[B64] Pan RR, Zhou LW (2016) *Hymenochaete conchata* sp. nov. (*Hymenochaetaceae*, *Basidiomycota*) from Chiang Mai, Thailand. Phytotaxa 273(3): 200–206. 10.11646/phytotaxa.273.3.7

[B65] Park JH, Pavlov IN, Kim MJ, Park MS, Oh SY, Park KH, Fong JJ, Lim YW (2020) Investigating wood decaying fungi diversity in Central Siberia, Russia using ITS sequence analysis and interaction with host trees. Sustainability 12(6): 2535. 10.3390/su12062535

[B66] Parmasto E (2001) Hymenochaetoid fungi (*Basidiomycota*) of North America. Mycotaxon 79: 107–176. 10.5962/p.418262

[B67] Parmasto E, Saar I, Larsson E, Rummo S (2014) Phylogenetic taxonomy of *Hymenochaete* and related genera. Mycological Progress 13: 55–64. 10.1007/s11557-013-0891-9

[B68] Popa F, Hertenstein A, Blumenstein K, Gantert LMR, Ordynets O, Rexer KH, Spirin V (2024) *Steccherinum pudorinum* comb. nov. Taxonomie und Ökologie einer Missdeuteten Art. Zeitschrift für Mykologie 90: 63–81.

[B69] Rambaut A (2018) FigTree. Tree Figure Drawing Tool, Version 1.4.4. University of Edinburgh, Edinburgh. http://tree.bio.ed.ac.uk/software/figtree/

[B70] Ronquist F, Huelsenbeck JP (2003) MrBayes 3: Bayesian phylogenetic inference under mixed models. Bioinformatics 19: 1572–1574. 10.1093/bioinformatics/btg180 12912839

[B71] Ronquist F, Teslenko M, van der Mark P, Ayres DL, Darling A, Höhna S, Larget B, Liu L, Suchard MA, Huelsenbeck JP (2012) MrBayes 3.2: Efficient Bayesian phylogenetic inference and model choice across a large model space. Systematic Biology 61: 539–542. 10.1093/sysbio/sys029 PMC3329765 22357727

[B72] Rossi W, Das K, Hembrom ME, Santamaria S, Parihar A, Ghosh A, Henkel TW, Hofstetter V, Randrianjohany E, Vizzini A, Wang XH, Buyck B (2020) Fungal Biodiversity Profiles 91–100. Cryptogamie Mycologie 41(4): 69–107. 10.5252/cryptogamie-mycologie2020v41a4

[B73] Ryvarden L (1991) Genera of polypores: Nomenclature and taxonomy. Synopsis Fungorum 5: 1–363.

[B74] Ryvarden L (2024) Hydnoid genera – a world synopsis. Synopsis Fungorum 50. Fungiflora, Oslo, 70 pp.

[B75] Song CG, Xu TM, Xu YH, Wang D, Zeng L, Fan XP, Sun YF, Cui BK (2025) Systematic revision, molecular phylogeny and divergence times of *Thelephorales (Basidiomycota)*. Mycosphere 16(1): 296–422. 10.5943/mycosphere/16/1/5

[B76] Spirin V, Runnel K, Põldmaa K (2015) Studies in the bark-dwelling species of *Hymenochaete* (*Hymenochaetales*, *Basidiomycota*) reveal three new species. Cryptogamie Mycologie 36: 167–176. 10.7872/crym/v36.iss2.2015.167

[B77] Stamatakis A (2014) RAxML version 8: A tool for phylogenetic analysis and post-analysis of large phylogenies. Bioinformatics 30: 1312–1313. 10.1093/bioinformatics/btu033 PMC3998144 24451623

[B78] Su JQ, Zhang XC, Zhao CL, Zhou HM (2024) Molecular phylogeny and morphology reveal a new wood-inhabiting fungal species, *Hyphoderma guangdongense* (*Polyporales*, *Basidiomycota*), from China. Phytotaxa 661: 75–86. 10.11646/phytotaxa.661.1.5

[B79] Sun YF, Xing JH, He XL, Wu DM, Song CG, Liu S, Vlasák J, Gates G, Gibertoni TB, Cui BK (2022) Species diversity, systematic revision and molecular phylogeny of *Ganodermataceae* (*Polyporales*, *Basidiomycota*) with an emphasis on Chinese collections. Studies in Mycology 101: 287–415. 10.3114/sim.2022.101.05 PMC9365044 36059897

[B80] Tellería MT, Dueñas M, Beltrán-Tejera E, Rodríguez-Armas JL, Martín MP (2012) A new species of *Hyphoderma* (*Meruliaceae*, *Polyporales*) and its discrimination from closely related taxa. Mycologia 104: 1121–1132. 10.3852/11-344 22495444

[B81] Tepin R, Singh RK (2025) *Hymenochaete liyiriana* sp. nov. (*Hymenochaetaceae*, *Basidiomycota*) from Arunachal Pradesh, Northeast India. New Zealand Journal of Botany 63(5): 1166–1176. 10.1080/0028825X.2024.2435962

[B82] Vinjusha N, Kumar TKA (2024) The *Hymenochaetales* of Kerala. Spore Print Books, Calicut, Kerala, 89 pp.

[B83] Vu D, Groenewald M, De Vries M, Gehrmann T, Stielow B, Eberhardt U, Verkley GJM (2019) Large-scale generation and analysis of filamentous fungal DNA barcodes boosts coverage for kingdom fungi and reveals thresholds for fungal species and higher taxon delimitation. Studies in Mycology 92: 135–154. 10.1016/j.simyco.2018.05.001 PMC6020082 29955203

[B84] Wagner T, Fischer M (2002) Proceedings towards a natural classification of the worldwide taxa *Phellinus* s.l. and *Inonotus* s.l., and phylogenetic relationships of allied genera. Mycologia 94: 998–1016. 10.1080/15572536.2003.11833156 21156572

[B85] Wallroth FG (1833) Flora Cryptogamica Germaniae, Pars II. Schrag, Nürnberg.

[B86] Wang L, Su JQ, Muhammad A, Zhao CL (2024) Two new wood-inhabiting fungal species (*Polyporales*, *Basidiomycota*) from Yunnan Province, China. Phytotaxa 647: 1–18. 10.11646/phytotaxa.647.1.1

[B87] Wang L, He SY, Lambert C, Zhu YG, Zhang JL, Yang Y, Li WL, Zhou HM, Zhao CL (2026) Comprehensive morphological and phylogenetic analyses of *Hymenochaetales (Basidiomycota)* unveil one new genus and twenty-six novel wood-inhabiting species from southwestern China. Persoonia 56: 328–380. 10.3114/persoonia.2026.56.05 PMC13409181 42524241

[B88] Wang XH, Das K, Bera I, Chen YH, Bhatt RP, Ghosh A, Hembrom ME, Hofstetter V, Parihar A, Vizzini A, Xu TM, Zhao CL, Buyck B (2019) Fungal Biodiversity Profiles 81–90. Cryptogamie Mycologie 40(5): 57–95. 10.5252/cryptogamie-mycologie2019v40a5

[B89] Westphalen MC, Rajchenberg M, Tomšovský M, Gugliotta AM (2018) A re-evaluation of Neotropical *Junghuhnia* s. lat. (*Polyporales*, *Basidiomycota*) based on morphological and multigene analyses. Persoonia 41: 130–141. 10.3767/persoonia.2018.41.07 PMC6344817 30728602

[B90] Westphalen MC, Motato-Vásquez V, Tomšovský M, Gugliotta AM (2021) Additions to the knowledge of hydnoid *Steccherinaceae*: *Cabalodontia*, *Etheirodon*, *Metuloidea*, and *Steccherinum*. Mycologia 113: 791–806. 10.1080/00275514.2021.1894536 34106041

[B91] Westphalen MC, Minosso NMM, Regio NC, Gugliotta AM, Rajchenberg M, Silveira RMB (2026) Unveiling the hidden diversity of neotropical *Steccherinum* and allied genera (*Steccherinaceae*, *Basidiomycota*). IMA Fungus 17: e182915. 10.3897/imafungus.17.182915 PMC12966849 41798708

[B92] White TJ, Bruns T, Lee SB, Taylor JW (1990) Amplification and direct sequencing of fungal ribosomal RNA genes for phylogenetics. In: Innis MA, Gelfand DH, Sninsky JJ, White TJ (Eds) PCR Protocols: A Guide to Methods and Applications. Academic Press, New York, 315–322. 10.1016/B978-0-12-372180-8.50042-1

[B93] Wijesinghe SN, Deng YL, Yuan Q, Zhou HM, Wang L, Dai YF, Xu Y, Jiang N, Al-Otibi F, Al-Sadi AM, Cao B, Chen Q, de Silva NI, Dong JH, Dong W, Du TY, Gu ZR, He SY, Jiang QQ, Li F, Li Q, Li W, Liu P, Liu WX, Liu XY, Liu ZB, Lu WH, Maharachchikumbura SSN, Su JQ, Tang SM, Tennakoon DS, Wang XC, Xiao WY, Xie ML, Yang X, Yang Y, Yang Yu, Yang YH, Yang YT, Zhang JL, Zhang SC, Zhang XC, Zhang XJ, Zhou Q, Al-Kharousi M, Al-Maqbali D, Al-Owaisi A, Al-Yahya’ei MN, Han XX, Hongsanan S, Hussain S, Jin JL, Karunarathna SC, Li CT, Li SL, Li Y, Luo ZL, Manawasinghe IS, Peng LY, Thongklang N, Tian WH, Tibpromma S, Velazhahan R, Xiao YP, Zhang ZJ, Zhao HJ, Zhao RL, Rathnayaka AR, Wanasinghe DN, Hyde KD, Zhao CL (2025) Mycosphere Notes 572–624: Exploring the hidden diversity of fungi and fungi-like taxa in different terrestrial microhabitats. Mycosphere 16(1): 2975–3129. 10.5943/mycosphere/16/1/21

[B94] Wu F, Zhou LW, Vlasák J, Dai YC (2022) Global diversity and systematics of *Hymenochaetaceae* with poroid hymenophore. Fungal Diversity 113: 1–192. 10.1007/s13225-021-00496-4

[B95] Wu G, Li YC, Zhu XT, Zhao K, Han LH, Cui YY, Li F, Xu JP, Yang ZL (2016) One hundred noteworthy boletes from China. Fungal Diversity 81: 25–188. 10.1007/s13225-016-0375-8

[B96] Wu SH (1997) New species of *Hyphoderma* from Taiwan. Mycologia 89(1): 132–140. 10.1080/00275514.1997.12026764

[B97] Wu YX, Dong JH, Zhao CL (2021a) *Steccherinum puerense* and *S. rubigimaculatum* spp. nov. (*Steccherinaceae*, *Polyporales*), two new species from southern China. Nova Hedwigia 113: 243–258. 10.1127/nova_hedwigia/2021/0636

[B98] Wu YX, Wu JR, Zhao CL (2021b) *Steccherinum tenuissimum* and *S. xanthum* spp. nov. (*Polyporales*, *Basidiomycota*): New species from China. PLOS ONE 16: e0244520. 10.1371/journal.pone.0244520 PMC7806176 33439872

[B99] Xu TM, Wu DM, Gao N, Liu S, Sun YF, Cui BK (2025) Species diversity, taxonomic classification and ecological habits of polypore fungi in China. Mycology 16(2): 419–544. 10.1080/21501203.2024.2384567 PMC12096708 40415919

[B100] Xu XM, Li M, Feng J, Chen M, Deng YL, Zhao CL, Shen S (2026) *Hymenochaete yaoshanensis* sp. nov. (*Hymenochaetaceae*, *Hymenochaetales*), a wood-inhabiting fungus from Yunnan Province, southwest China. Phytotaxa 755(2): 154–174. 10.11646/phytotaxa.755.2.2

[B101] Yang J, He SH (2014) *Hymenochaete* in China. 8. *H. biformisetosa* sp. nov. with a key to species with denticulate setae. Mycotaxon 128(1): 137–144. 10.5248/128.137

[B102] Yang JW, Cha L, Cheng LJ, Yang SQ, Zhao CL, Zhou HM (2023) Molecular systematics and taxonomic analyses of three new wood-inhabiting fungi of *Hyphoderma* (*Hyphodermataceae*, *Basidiomycota*). Journal of Fungi 9: 1044. 10.3390/jof9111044 PMC10672532 37998850

[B103] Yang Y, Xu Y, Wang L, Jiang QQ, Su JQ, Li R, Zhou HM, Zhao CL (2025) Multigene phylogeny of seven wood-inhabiting fungal orders in *Basidiomycota*, and proposal of a new genus and thirteen new species. Mycosphere 16: 245–295. 10.5943/mycosphere/16/1/4

[B104] Yuan HS, Dai YC (2008) Polypores from northern and central Yunnan Province, Southwestern China. Sydowia 60: 147–159.

[B105] Yuan HS, Wu SH (2012) Two new species of *Steccherinum* (*Basidiomycota*, *Polyporales*) from Taiwan. Mycoscience 53(2): 133–138. 10.1007/S10267-011-0139-Y

[B106] Yuan HS, Lu X, Qin WM (2019) Molecular and morphological analyses separate *Junghuhnia pseudocrustacea* sp. nov. (*Basidiomycota*) from *Junghuhnia crustacea* complex. Nova Hedwigia 108: 255–264. 10.1127/nova_hedwigia/2018/0497

[B107] Yuan HS, Zhou LJ, Zhu YQ, Wei YL, Zhang XJ, Zhao CL, Zhao H (2026) Species diversity of corticioid and hydnoid fungi in China and their medicinal, environmental, agricultural and industrial values. Mycosphere 17: e005. 10.48130/mycosphere-0026-0004

[B108] Yurchenko E, Wu SH (2014a) *Hyphoderma pinicola* sp. nov. of *H. setigerum* complex (*Basidiomycota*) from Yunnan, China. Botanical Studies 55: 71. 10.1186/s40529-014-0071-5 PMC5430344 28510951

[B109] Yurchenko E, Wu SH (2014b) *Hyphoderma formosanum* sp. nov. (*Meruliaceae*, *Basidiomycota*) from Taiwan. Sydowia 66: 19–23. 10.12905/0380.sydowia66(1)2014-0019

[B110] Yurchenko E, Wu SH (2015) *Hyphoderma moniliforme* and *H. nemorale* (*Basidiomycota*) newly recorded from China. Mycosphere 6: 113–121. 10.5943/mycosphere/6/1/11

[B111] Yurchenko E, Larsson KH, Riebesehl J, Miettinen O (2023) *Steccherinum filiferum* sp. nov. from the Neotropics, and a new combination for *Odontia laxa*. Mycotaxon 137: 773–786. 10.5248/137.773

[B112] Zhang QY, Liu HG, Papp V, Zhou M, Dai YC, Yuan Y (2023) New insights into the classification and evolution of *Favolaschia* (*Agaricales*, *Basidiomycota*) and its potential distribution, with descriptions of eight new species. Mycosphere 14: 777–814. 10.5943/mycosphere/14/1/10

[B113] Zhang XJ, Wang JY, Yang RC, Zhang SH, Zhang SC, Xu J, Zhao CL (2025) Morphological characteristics and phylogenetic analyses reveal *Steccherinum farinaceum* sp. nov. (*Polyporales*, *Basidiomycota*) from Yunnan Province, China. Phytotaxa 722: 16–28. 10.11646/phytotaxa.722.1.2

[B114] Zhao CL, Cui BK, Song J, Dai YC (2015) *Fragiliporiaceae*, a new family of *Polyporales (Basidiomycota)*. Fungal Diversity 70: 115–126. 10.1007/s13225-014-0299-0

[B115] Zhao H, Cui YJ, Guan QX, Wang K, Zhuang L, Zeng GY, Wei YL, Wu F, Yuan HS (2025) Global species diversity and distribution patterns within the order *Hymenochaetales* (*Agaricomycetes*, *Basidiomycota*). Mycosphere 16(1): 3257–3280. 10.5943/mycosphere/16/1/24

[B116] Zhou M, Dai YC, Vlasák J, Liu HG, Yuan Y (2023) Updated systematics of *Trichaptum* s.l. (*Hymenochaetales*, *Basidiomycota*). Mycosphere 14: 815–917. 10.5943/mycosphere/14/1/11

[B117] Zhu YQ, Zhou LJ, Liu JX, Yuan YW, Wei Y, Wei YL, Yuan HS (2026) Thirty new species in *Thelephorales* and *Hymenochaetales* from the Eastern Himalayas and Southern China. Mycosphere 17: e001. 10.48130/mycosphere-0026-0001

[B118] Zmitrovich IV, Kovalenko AE (2016) Lentinoid and polyporoid fungi, two generic conglomerates containing important medicinal mushrooms in molecular perspective. International Journal of Medicinal Mushrooms 18: 23–38. 10.1615/IntJMedMushrooms.v18.i1.40 27279442

